# The use of implementation science theoretical approaches in hybrid effectiveness-implementation type 1 randomised trials of healthcare interventions: A scoping review

**DOI:** 10.1186/s13012-025-01435-6

**Published:** 2025-05-16

**Authors:** Orly Atzmon, Meagan E. Crowther, Bei Bei, Denise A. O’Connor

**Affiliations:** 1 School of Psychological Sciences, Monash University, Melbourne, Australia; 2 School of Public Health and Preventive Medicine, Monash University, Melbourne, Australia

**Keywords:** Hybrid type 1 trial, Hybrid type 1 effectiveness-implementation randomised controlled trial, Implementation science, Theory, Model, Framework

## Abstract

**Background:**

Hybrid type 1 effectiveness-implementation randomised controlled trials (RCTs) aim to accelerate the translation of proven clinical interventions into routine care by concurrently investigating the effectiveness of clinical interventions and the context for real-world implementation. Hybrid type 1 RCTs can make use of implementation science theoretical approaches (i.e., theories, models, and frameworks) to understand barriers and facilitators to sustainable implementation of clinical interventions; however, the extent to which these approaches have been used in hybrid type 1 RCTs has not been systematically investigated. This scoping review aimed to investigate the extent to which implementation science theoretical approaches have been used in hybrid type 1 RCTs of healthcare interventions and describe which approaches have been reported and how they have been used.

**Methods:**

The review was conducted in accordance with the pre-registered protocol (https://doi.org/10.17605/OSF.IO/CJ8A7). Searches of six electronic databases were conducted for published hybrid type 1 RCTs evaluating any clinical intervention in any healthcare setting. The included trials were full-text, peer-reviewed primary research articles written in English, and reporting the findings of hybrid type 1 RCTs of healthcare interventions. Non-English language reports, reviews, protocols without a linked trial results report, methodological papers, opinion pieces, commentaries, books/book chapters, dissertations, and conference abstracts were excluded. Two reviewers independently selected studies, extracted data, and assessed use of theoretical approach/es.

**Results:**

We identified 8,878 citations, screened 673 full-text records, and included 37 trials.

Most trials were conducted in North America (68%), investigating clinical interventions for mental health problems (32%) in adults (43%). Twenty-eight (76%) trials cited use of at least one theoretical approach. The most common was the Reach, Effectiveness, Adoption, Implementation, and Maintenance (RE-AIM) framework (43%). Theoretical approaches were most often applied (62%) to justify the implementation study design, guide selection of study materials or analyse implementation outcomes.

**Conclusion:**

The majority of published hybrid type 1 effectiveness-implementation RCTs of healthcare interventions report using at least one theoretical approach to explore the context for implementation. Use of implementation science theories, models, and/or frameworks to understand the barriers and facilitators to implementation and sustainability of proven clinical interventions is likely to accelerate future translation of evidence-based practices into routine care and thus optimise patient outcomes.

**Supplementary Information:**

The online version contains supplementary material available at 10.1186/s13012-025-01435-6.

Contributions to the literature

- This scoping review investigates and synthesises evidence on the usage of implementation science theories, models, and frameworks in hybrid type 1 effectiveness-implementation randomised controlled trials of healthcare interventions.
- By investigating to what extent and how these theories, models, and frameworks are used, this review offers valuable insights for future researchers in selecting appropriate theoretical approaches to explore the context for sustainable implementation of proven clinical interventions into routine care.
- Theory-informed assessments of barriers and facilitators to uptake of evidence-based clinical interventions informs the design of tailored implementation strategies, which may accelerate translation into routine practice.

## Background

The traditional research trajectory to support translation of evidence-based clinical interventions into routine practice for improved patient care has emphasised a staged approach. This staged approach creates a time lag as it focusses on first establishing that a clinical intervention works under ideal conditions (i.e., performs well in efficacy and subsequent effectiveness trials and systematic reviews) before considering translation into routine practice [1]. More recently, this approach also includes de-implementation of clinical interventions that are demonstrated to be of little or no benefit, or are harmful to patients [2]. As a result, there is often a substantial time lag between the creation of an evidence-based clinical intervention and its widespread implementation within the community [3]. Hybrid effectiveness-implementation randomised controlled trials (RCTs) have been conceptualised to reduce this time lag and accelerate implementation of evidence-based clinical interventions into routine practice. Hybrid trial designs take a dual focus in assessing the effectiveness of a clinical intervention and its concurrent implementation [1]. Type 1 hybrid trials aim to concurrently investigate the effects of clinical interventions as well as the context for implementation. Type 2 hybrid trials assess the effects of clinical interventions while also exploring the feasibility and potential utility of implementation strategies to support their uptake. Type 3 hybrid trials assess the effects of implementation strategies on implementation as well as clinical outcomes [1].

There is value in using theories, models, and/or frameworks (TMFs) in implementation research [4]. TMFs can aide in exploring various factors influencing the implementation and sustainability of clinical interventions (e.g., barriers and facilitators), can inform the design of implementation strategies to support uptake of clinical interventions in routine health care, and facilitate evaluation of implementation outcomes [4, 5]. TMFs provide an understanding of the complex systems within which implementation occurs, and provide explicit assumptions that can be tested, validated, or refined in empirical studies [6]. TMFs also help to connect findings across studies from various clinical settings [7]. As such, they support efficiency in generalising knowledge across contexts, thereby advancing implementation science [6, 7]. Nilsen [4] has proposed a taxonomy of TMFs in implementation science (Table 1).

**Table 1:** Five categories of theories, models and frameworks used in implementation science

Category	Description
Process models	They specify steps (stages, phases) in the process of translating research into practice, including the implementation and use of research
Determinant frameworks	They specify types of determinants and individual determinants, which act as barriers and enablers that influence implementation outcomes
Classic theories	Theories that originate from fields external to implementation science, which can be applied to provide understanding and/or explanation of aspects of implementation
Implementation theories	Theories that have been developed by implementation researchers to provide understanding and/or explanation of aspects of implementation
Evaluation frameworks	They specify aspects of implementation that could be evaluated to determine implementation success

Table adapted from Nilsen [4]

Numerous TMFs are available [4, 8, 9]. Yet, a comprehensive analysis of 235 implementation studies by Davies et al. [10] shows that less than one quarter use TMFs in any way, and only 6% are explicitly theory-based. More recent reviews by Colquhoun et al. [11] and McIntyre et al. [12] highlight a similar trend, where only 14% of randomised trials of audit and feedback interventions report use of TMFs, and only a quarter of process evaluations conducted alongside implementation trials are informed by, apply, or test TMFs, respectively. Therefore, the use of TMFs in implementation trials, and accompanying process evaluations, is suboptimal.

Hybrid type 1 effectiveness-implementation RCTs can utilise TMFs to better understand and describe the context for implementation of clinical interventions. By examining barriers and facilitators to implementation and sustainability, TMFs can provide valuable insights to understand how clinical interventions may function effectively in specific contexts, thereby informing the development of tailored strategies for successful integration of the interventions into routine practice [7]. To our knowledge, the extent to which TMFs are used in hybrid type 1 effectiveness-implementation RCTs of healthcare interventions has not been systematically investigated. To fill this gap, a scoping review was conducted to investigate the extent to which hybrid type 1 effectiveness-implementation RCTs report using one or more TMFs to explore the implementation context, and to describe which theoretical approaches have been reported and how they have been used. The objectives of this review are to: (1) investigate the proportion of hybrid type 1 effectiveness-implementation RCTs of healthcare interventions that explicitly report using one or more TMFs; (2) describe which TMFs are reported; and (3) examine how the TMFs are used.

## Methodology

### Study design

A scoping review method was selected as it is particularly useful for systematically mapping findings across a body of evidence that is diverse and complex [13]. The review was conducted in accordance with the Joanna Briggs Institute methodology for scoping reviews which is based on Arksey and O'Malley [14] and Levac et al. [15]. There are six steps including: (1) defining the research question/s, (2) identifying relevant studies, (3) study selection, (4) charting the data, (5) collating, summarising, and reporting the results, and (6) consultation. The present review is reported in line with Preferred Reporting Items for Systematic Reviews and Meta-Analysis extension for Scoping reviews (PRISMA-Scr) guidelines [16] (Additional File 1).

### Protocol and registration

The scoping review protocol was pre-registered on January 19, 2024 (Open Science Framework: 10.17605/OSF.IO/CJ8A7). Prior to protocol registration, one author searched the Cochrane Database of Systematic Reviews, PROSPERO, and OSF, and determined that no similar reviews were already underway. The following sections describe the steps taken for conducting this scoping review. Quality appraisal was not undertaken as this review aimed to map whether and how TMFs are used in hybrid type 1 effectiveness-implementation RCTs rather than to synthesise the effects of clinical interventions, therefore, a quality assessment was not considered relevant.

## Eligibility criteria

### Types of studies

Studies were eligible for inclusion if they were hybrid type 1 effectiveness-implementation RCTs investigating the effectiveness of a healthcare clinical intervention while also exploring the context for implementation [1]. The included trials were full-text, peer-reviewed primary research articles written in English, and reporting the findings of a hybrid type 1 effectiveness-implementation RCTs. Non-English language reports, as well as reviews, protocols without a linked trial results report, methodological papers, opinion pieces, commentaries, books or book chapters, dissertations, and conference abstracts were excluded. Hybrid type 2 and 3 RCTs were not within the scope of the funded project and hence excluded. Date limits were set from when the effectiveness-implementation hybrid typology was published in March 2012 [1] as hybrid trials were not defined in research before this time. Multiple reports of the same trial found within our search, including publications such as protocols, reports of trial results, and qualitative or mixed-method reports, were collated so that each trial, rather than each report, was the unit of interest in the review.

### Types of participants

The population of interest were participants who received the clinical interventions of interest in the hybrid type 1 effectiveness-implementation RCTs, healthcare providers who delivered the interventions, and/or stakeholders who were or would be interested in the interventions.

### Types of interventions and settings

This review considered hybrid type 1 effectiveness-implementation RCTs that evaluated any clinical intervention in any healthcare setting. A healthcare intervention is defined as an intervention carried out to improve, maintain, or assess the health of a person in a clinical situation [17, 18]. An intervention that provided preventative intervention where a clinical condition was not present were excluded. Interventions that are not clinical interventions delivered in a healthcare setting were excluded. A healthcare setting includes hospitals (inpatient or outpatient care, acute or subacute phase), primary care, residential care, and the community. Excluded settings were where healthcare was not the primary function (e.g., correction facilities and services, community senior centres and schools).

### Types of outcomes

Included trials explored the context for implementation and/or sustainability of the clinical intervention, including barriers and facilitators. Barriers refer to any factors reported to impede implementation efforts, and facilitators are any factors which enable implementation.

### Search strategy

A two-step search was utilised in this review. The first step involved using search terms to retrieve hybrid type 1 effectiveness-implementation RCTs in six electronic databases (Ovid Medline, Ovid EMBASE, PsycINFO, CINAHL (EBSCO), Cochrane CENTRAL and Scopus). The second step involved citation tracking in Scopus of the original paper by Curran et al. [1] describing the effectiveness-implementation hybrid design typology (Additional File 2).

The search strategy in step one was developed in consultation with an academic librarian at Monash University. The search strategy was adapted for each chosen database, and Boolean operators and relevant controlled vocabulary terms were used as needed for each database search. RCT filters utilised in the search strategy were based on those reported in the Cochrane Handbook for Systematic Reviews of Interventions version 6.4 [19] for Medline (Ovid) and the Cochrane Embase RCT filters for Ovid, PsycINFO search strategy published by Eady et al. [20], and the Cochrane RCT filter for CINAHL Plus [21]. Initial database searches were conducted on 31st October 2023, with a final search performed on 26th November 2024 to identify any additional publications released between 31st October 2023 and 26th November 2024. All search results were managed in EndNote [22] and then exported to Covidence [23].

### Selection of sources of evidence

Following deduplication, two reviewers (OA, MC) independently screened all titles and abstracts identified in the search according to the eligibility criteria. Following this, the same reviewers independently screened the full text of all potentially eligible records, recording reasons for exclusion of ineligible trials. Any disagreements in the screening and selection process were resolved through discussion between the two reviewers and, where needed, adjudication by a third reviewer (DO). Authors were contacted if clarification was needed for whether a publication was part of a trial. Additionally, reviewers carefully examined the aims and methods of trial reports to ensure that trials were hybrid type 1 effectiveness-implementation RCTs if they did not specify within publications. The search and screening results were summarised using a PRISMA flow chart.

### Data charting process

Two reviewers (OA, MC) independently extracted data from the included trials using a standardised data extraction form developed by the research team (Additional File 3). Any disagreements were resolved through discussion between the reviewers and, where needed, adjudication by a third reviewer (DO). Prior to completing data extraction, the data extraction form was independently piloted by the two reviewers on 10 randomly selected publications. The pilot data extraction was then compared, discussed and the form refined to ensure consistency in content extracted.

### Data items

#### Category 1: Trial characteristics

The following characteristics were extracted: publication title, year of publication, first author, source of publication (journal), country, trial aim/s, trial setting, type/s and number of participants in the effectiveness component of the trial, description of the clinical intervention, data collection and method/s for the implementation component of the trial, type/s and number of participants included in the implementation component of the trial.

#### Category 2: What TMFs were used

Data extracted included the name/s, source/s (if provided), type/s (Table 1) and number of TMFs cited. Reviewers categorised TMFs based on their alignment with Nilsen’s [4] categories.

#### Category 3: How TMFs were used

The extent to which each TMF was used in the implementation component of the trial was categorised as one or more of the following: “informed by”, “applied” or “cited only”. Our scheme followed an adapted version of Painter et al. [24] classification, utilising the original “informed by” and “applied” categories, and excluding “testing theory” and “building or creating theory”, as these are beyond the scope of hybrid type 1 effectiveness-implementation trials. Additionally, we added a “cited only” category to reflect trials that only cited a theory with no additional explanation or elaboration of how it was used [12]. “Applied” use was operationalised as the theoretical approach being specified with approximately half or more of the constructs applied in the implementation component of the trial (e.g., a trial described use of a theoretical approach to develop data collection tools (e.g., survey, interview topic guide) and the constructs were measured in the tools). “Informed by” was operationalised as the theoretical approach being specified with limited application within trial components and measures (e.g., a trial described use of a theoretical approach to inform qualitative data analysis but there was no evidence of the constructs being explored or examined). “Cited” was when a theoretical approach was mentioned or referenced in a trial without any further elaboration on how it was applied. Reviewers employed a descriptive approach to analyse qualitative data, further elaborating on the utilisation of TMFs. Such analysis aimed to ascertain the degree to which TMFs were applied or employed, facilitating a comprehensive evaluation of their usage. This analysis used themes adapted from McIntrye al’s [12] categorisation for TMFs utilisation in process evaluations and Colquhoun et al. [11] category of justification of TMFs use for audit and feedback, both modified to fit the context of hybrid type 1 effectiveness-implementation trials. For trials that cited multiple TMFs, use was assessed for each individually.

### Data synthesis

Extracted data were analysed to calculate the proportion of hybrid type 1 effectiveness-implementation RCTs utilising TMFs. Data analysis included frequency counts to identify the number of times each approach was used according to the use categories. The data synthesis was conducted by one reviewer (OA) and checked by a second reviewer (MC).

## Results

### Selection of sources of evidence

**Fig. 1:**
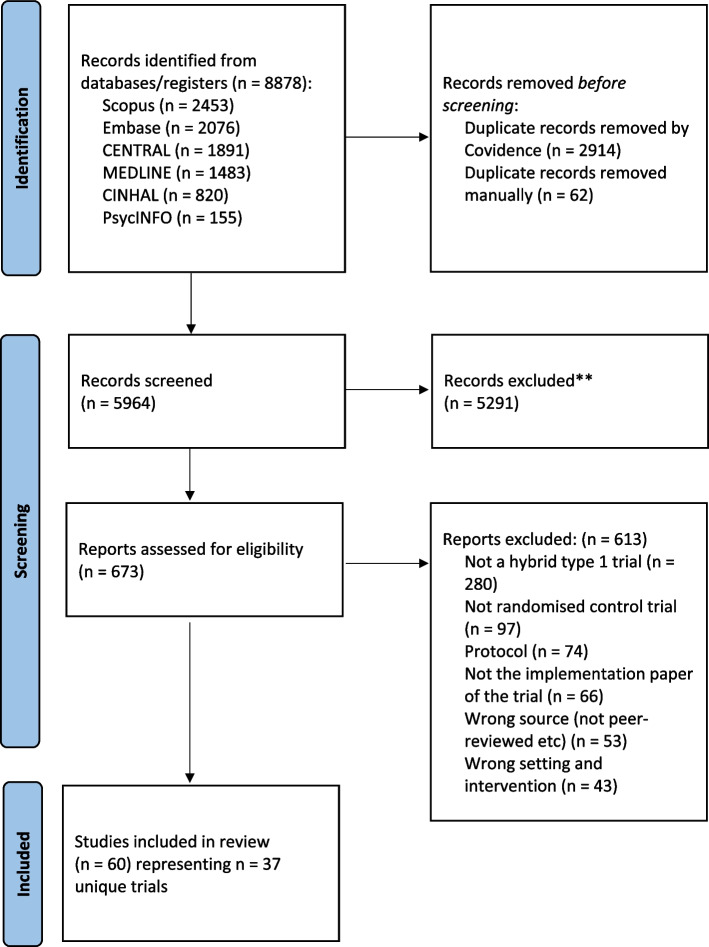
The PRISMA flowchart

Database searches until the 26th of November 2024 retrieved a total of 8,878 records. After removing duplicates, 5,964 records remained for screening. Following title and abstract eligibility screening, 673 records remained for full-text review. Of these, 613 records were excluded for various reasons, including being protocols (*n* = 74; 12%), not being hybrid type 1 effectiveness-implementation designs (*n* = 280; 46%), and not being a RCT (*n* = 97; 16%). Clarification of the eligibility of 23 records was provided by a third reviewer (DO; Additional File 4). A total of 60 publications were eligible for inclusion in the review (Fig. 1), which represented 37 unique hybrid type 1 effectiveness-implementation RCTs.

### Characteristics of included trials

A summary of the trial characteristics is provided in Additional file 5. Geographically, the studies were predominantly conducted in North America (*68%*), then Africa (*14%*), Europe (*11%*), Australia and New Zealand (*5%*), and Central and South America (*3%*). Intervention settings were primarily community-based (*46%*), with a smaller number conducted in primary care (*24%*), Veterans’ affairs settings (*19%*), hospitals (*5%*), and nursing homes *(5%).* The target populations varied, with *43%* of trials focussing on adults, *11%* on women only, *11%* on veterans, *8%* on children, *8%* on older adults and *3%* on men only. A notable portion of included trials did not describe characteristics of the trial participants (*16%*). The majority (*32%*) of trials assessed the effects of clinical interventions for mental health problems, substance use problems (*16%*) and diabetes (*14%*).

### Implementation characteristics

Most trials assessed implementation outcomes using a combination of interviews and questionnaires/checklists (*n* = 10; *27%*), or interviews alone (*n* = 9; *24%*). Questionnaires or checklists alone were used in four trials (*11%*), and chart reviews or report reviews were used in three trials (*8%*; Additional file 5). Participants in the implementation aspect of the trials comprised of intervention providers (*n* = 10; *29%*), those who received the intervention in the effectiveness aspect of the trial (*n* = 6; *17%*), relevant stakeholders (*n* = 4; *11%*), and various combinations of these groups (*43%*; Additional File 5).

## Synthesis of results

### What proportion of trials cited TMFs and how were they used?

Of the 37 [25–83] included trials, 28 [25–70] (*76%*) used at least one TMF related to implementation, with 9 of these trials [25–34, 69] using two theoretical approaches, and two trials [35–40] utilising three. At least one TMF was applied in 23 (62%) of the 28 trials, informed seven (19%) trials and was cited in two (5%) trials. Nine trials (*24.3%*) did not use any theoretical approach [71–83]. Overall, a theoretical approach was used 41 times across the 28 trials (see Table 2).

**Table 2:** Extent to which theories, models and frameworks were used in included trials

	**Frequency* (%)***	**Trial citations**
TMFs used	41	
Applied	32 *(78.0)*	[25–35, 37, 39–47, 49–53, 60, 69]
Informed by	7* (17.1)*	[26, 33, 48, 54, 55, 69, 70]
Cited	2* (4.9)*	[27, 50]

Trials may appear in multiple categories if they employed more than one theoretical approach, with each theory, model, or framework assessed individually. Additionally, only the implementation studies of the trials are referenced

An analysis of the qualitative descriptions provided by trialists on how, and the extent to which, TMFs were used in the trials was conducted and is presented in Table 3 and Additional File 6 respectively. Most commonly, theoretical approaches were ‘applied’ to justify the design, select study materials, or analyse data (*n* = 23; *62%*). For example, Arrossi et al. [37] used the Reach, Effectiveness, Adoption, Implementation, and Maintenance (RE-AIM) framework in all stages of the research process, including conceptualisation, data collection, and analysis whilst Woodard et al. [41] used RE-AIM to evaluate implementation outcomes across its dimensions, specifically reach, adoption and implementation (see Additional File 6).

**Table 3:** How theories, models and frameworks were used in included trials

	N (*%*)
Applied^a^	32/41
To select study materials and analyse data	17(*41.5)*
To select study materials/design data collection	8(*19.5*)
To analyse data	2 (*4.9)*
To justify the study design and select study materials	3(*7.3)*
To justify the study design, select study materials and analyse data	1(*2.4)*
To justify the study design	1(*2.4)*
Informed by^a^	7/41
To support the implementation-related aims/objectives	4*(9.8)*
To describe/explain the results	1(*2.4)*
To support the implementation-related aims/objectives and describe/explain the results	1(*2.4)*
Informed intervention	1(*2.4*)

^a^Related to implementation component of trial

Numerous TMFs were used to develop implementation study materials, including interview or focus group topics guides [25, 34–36, 42–47] and questionnaire items [48]. Many TMFs also guided other data collection methods (i.e., chart reviews, intervention cost tracking, surveys, rating scales) [28, 29, 39, 49–51]. Additionally, RE-AIM and the Consolidated Framework for Implementation Research (CFIR) were used most frequently to analyse qualitative and/or quantitative data [26, 33, 34, 40, 46, 52, 69].

Less than one quarter of trials were ‘informed by’ one or more theoretical approaches to support the aims of the implementation component of the trial or to explain the results (*n* = 8; *22%*). For example, two trials reported their trial hypothesis was informed by the Exploration, Preparation, Implementation and Sustainment (EPIS) [70] model and the RE-AIM framework [53]. Other trials indicated that a theoretical approach informed the interpretation of the implementation components of the trial, with Frost et al. [54] using Social Practice Theory, Petersen et al. [55] using RE-AIM, and Teupen et al. [69] using Grant et al.’s [84] framework for process evaluations of cluster randomised trials. Additionally, Chlebowski et al. [27] employed the Framework for Reporting Adaptations and Modifications-Enhanced (FRAME) to interpret their trial results. Another study by Minian et al. [33] utilised the Interactive Systems Framework (ISF) to guide the delivery of the intervention. Lastly, two trials only cited a theoretical approach [27, 50].

Of the 11 trials that utilised more than one TMF, two trials utilised three TMFs and both trials *applied* all TMFs with one trial applying RE-AIM, COM-B, and Self-Determination Theory to select study materials and analyse data [35, 36] and the other trial applying RE-AIM for the selection of study materials, analysis of data, and justification of the study design, while Proctor’s Taxonomy was used to select study materials and CFIR was used to select materials and analyse data [37–40] (see Additional File 6). For studies that utilised two TMFs (9 trials), three trials applied both TMFs for the same purpose, with one trial using two TMFs to select study materials and analyse data [25], while the other two trials applied two TMFs to select study materials [28, 34]. Other trials *applied* both theoretical approaches in different ways. For instance, Proctor’s taxonomy of implementation outcomes was used to justify the study design and select study materials, while RE-AIM was applied to select study materials and analyse the data [29, 30]. Another trial applied RE-AIM for selecting the study design and materials, while iPARIHS was used to justify the study design and select materials [31, 32]. Interestingly, four trials [27, 33, 69] applied one theoretical approach while being informed by or citing another.

### Which TMFs were reported?

The most commonly reported theoretical approach was the RE-AIM framework, appearing in 12 out of 28 (*43%*) trials (see Table 4). This was followed by the CFIR, which was used in nine (*32%*) trials, and Proctor’s taxonomy of implementation outcomes, used in four (*14%*) trials. Overall, evaluation frameworks were the most frequently employed theoretical approaches.

**Table 4:** TMFs reported in included trials

TMF category	TMF name (citation)	N (*%*) used	Trial citations
Evaluation frameworks			
	Reach, Effectiveness, Adoption, Implementation, and Maintenance (RE-AIM) [85]	*12 (42.9)*	[26, 28, 30, 31, 33, 34, 37, 40, 41, 43, 45, 53, 55]
	Proctor’s taxonomy of implementation outcomes[86]	*4 (14.3)*	[27, 29, 39, 50]
	Grant et al [84] framework for process evaluations of cluster randomised trials	*1 (3.6)*	[69]
	Practical Robust Implementation Sustainability Model (PRISM) [87]	*1 (3.6)*	[51]
Determinant frameworks			
	Consolidated Framework for Implementation Research (CFIR) [88]	*9 (32.1)*	[25, 26, 34, 40, 42, 46, 47, 52, 69]
	Integrated Promoting Action on Research Implementation in Health Services (iPARiHS) [89, 90]	*3 (10.7)*	[28, 32, 49]
	Exploration, Preparation, Implementation and Sustainment (EPIS) [91]	*1 (3.6) *	[70]
	Nonadaptation, Abandonment, and Challenges to the Scale-up, Spread and Sustainability of Health and Care Technologies (NASSS) framework [92]	*1 (3.6)*	[35]
	Practical Robust Implementation Sustainability Model (PRISM) [87]	*1 (3.6)*	[51]
	Socio-ecological model [93]	*1 (3.6)*	[44]
Classic theories			
	Theory of Diffusion of Innovations [94]	*2 (7.1)*	[25, 48]
	Self-determination theory [95]	*1 (3.6)*	[35]
	Social Practice Theory [96]	*1 (3.6)*	[54]
Process models			
	Exploration, Preparation, Implementation and Sustainment (EPIS) [91]	*1 (3.6) *	[70]
	Interactive Systems Framework (ISF) for dissemination and implementation [97]	*1 (3.6)*	[33]
Implementation theories			
	Capability, Opportunity, Motivation and Behaviour (COM-B) [98]	*1 (3.6)*	[35]
	Organisation Readiness for Change Theory[99]	*1 (3.6)*	[60]

Two theoretical approaches (EPIS and PRISM) are coded in two of Nilsen’s categories. Additionally, one framework (FRAME) could not be coded according to Nilsen’s taxonomy as it appears to provide a checklist to assess adaptations and modifications to interventions.

## Discussion

### Summary of evidence

This scoping review identified 60 publications reporting 37 hybrid type 1 effectiveness-implementation trials of healthcare interventions and examined the use of implementation science theoretical approaches in these studies. Three quarters of trials used at least one theoretical approach. Of these, nearly two thirds of studies ‘applied’ and nearly one quarter were ‘informed by’ one or more TMFs. Eleven trials used more than one TMF. The RE-AIM and CFIR frameworks were the most frequently used theoretical approaches. Evaluation frameworks, such as RE-AIM, were the most commonly used category of theoretical approach.

The finding that the majority of included trials utilised at least one theoretical approach is in contrast to McIntyre et al.’s [12] review in 2020 that found only 26% of studies utilised a TMF in process evaluations conducted alongside implementation trials. This suggests that the use of TMFs in trials may be increasing. This finding is a positive development, as the use of TMFs in hybrid effectiveness-implementation trials is likely to enhance the understanding of factors that may be important determinants of, and inform design of tailored implementation strategies to support, practice change if the clinical intervention is shown to be effective in the effectiveness component of the hybrid trial. The current review focussed on hybrid type 1 trials published since 2012, when Curran et al. [1] coined the term ‘hybrid trials’, whereas McIntyre et al. [12] focussed on process evaluations conducted alongside published implementation trials, and thus did not have a date limit. The inclusion of more recent trials in our review may be one possible explanation for the higher use of TMFs. This trend may also be influenced by recent efforts to categorise TMFs [4] and provide pragmatic guides for researchers on how to utilise TMFs in implementation projects [100, 101]. An additional potential explanation for the increased use of TMFs may be the recent emphasis on incorporating TMFs in grant proposals for implementation research [102, 103].

Our review highlighted that hybrid type 1 RCTs can utilise implementation science frameworks in numerous ways to provide actionable insights to inform future implementation efforts, for example, by informing selection or design of data collection tools, evaluating implementation outcomes, and analysing qualitative and/or quantitative data. Evaluation frameworks specify aspects of implementation that could be evaluated to determine implementation success [4]. Our finding that RE-AIM, an evaluation framework, was the most common theoretical approach used within the included trials is interesting considering hybrid type 1 trials aim to investigate the context for implementation, including barriers and facilitators to change, rather than evaluating current implementation success. Considering this focus of hybrid type 1 trials, it is surprising that determinant frameworks (e.g., CFIR, PARiHS), which explicitly help in exploring the factors influencing implementation, were not more widely used. A possible explanation for this finding is that researchers conducting hybrid type 1 trials may not be as familiar with determinant frameworks and how they can help in exploring the factors influencing implementation of clinical interventions. Nonetheless, a scoping review of the usage of TMFs in implementation science found that determinant frameworks were the most commonly used [104], therefore the limited use of determinant frameworks could be due to other factors, which future research could explore. In addition to the CFIR, there are other determinant frameworks which were not reported in the included trials in our review but are likely to provide a useful basis for theorising the pathways to implementation of new evidence-based clinical practices (e.g., the Theoretical Domains Framework and the Model for Diffusion of innovations in Service Organisations) [101, 105–107].

Numerous implementation TMFs are available, making the selection process potentially challenging for researchers [108]. Recent advancements in the field, such as new guidance for researchers in selecting TMFs based on the ‘goodness-of-fit’ between the aims of the study and the characteristics of theoretical approaches, may be useful moving forward. For example, Lynch and colleagues [100] have proposed five questions to consider when selecting theoretical approach(es): (i) who are you working with? (e.g., individuals, groups or wider settings); (ii) when in the process are you going to use theory? (i.e., are you planning, conducting or evaluating?); (iii) why are you applying a theory? (i.e., what is your aim and what do you need to understand?); (iv) how will you collect data? (e.g., routinely collected data or data informed by the theoretical approach?); and (v) what resources are available? The implementation Theory Comparison and Selection Tool (‘T-CaST’) [109] is also available, informed by surveys and interviews with 37 implementation scientists across USA, the UK and Canada and containing 16 items across 4 domains (usability, testability, applicability, acceptability) to inform theory selection. Additionally, there are several resources that can support learning about implementation theoretical approaches. Websites for RE-AIM and CFIR, for example, help researchers keep up-to-date with advances in these frameworks (e.g., https://re-aim.org, https://cfirguide.org), whilst text books also summarise numerous implementation TMFs [110–112]. Furthermore, it may be useful for researchers with expertise in implementation science to be included in hybrid trial study teams from early in the research process to enhance the rigor and relevance of the implementation aspects of these trials, ensuring that chosen TMFs are maximally useful and effectively applied.

Of the trials that utilised TMFs in our review, the majority ‘applied’ these approaches as opposed to the trials being ‘informed by’ them. Applying TMFs is considered a higher-level use than being informed by TMFs, with many trials applying them to guide data collection methods, develop interview guides and questionnaires, and analyse data. In contrast, trials that were ‘informed by’ one or more theoretical approaches used them primarily to support the aims of the implementation component of the trial, the interpretation of the implementation component, and for interpreting trial results. More trials ‘applying’ theoretical approaches suggests a deeper integration of implementation science principles into the design and execution of the implementation component of hybrid type 1 trials. Such integration of implementation science theoretical approaches provides a structured foundation for research, ensuring that studies are grounded in tested and validated concepts.

In the current review, many of the TMFs were challenging to categorise according to Nilsen’s taxonomy [4] due to inadequate trial explanations and reporting. Future trials should provide more detailed descriptions of the theoretical approach/es used, including a clear explanation of how they were utilised in the context of the trial. Additionally, there was heterogeneity in reporting between the different publications of trials (e.g., mentioning a theoretical approach in the protocol paper but not specifying it in the paper reporting implementation aspects), which made identifying, categorising and synthesising the use of TMFs challenging. In our scoping review we categorised approaches according to Nilsen’s [4] taxonomy of TMFs in implementation science to ensure consistency within the literature. This structured approach can contribute to more robust implementation science research, guiding researchers in selecting and applying the most appropriate TMFs in their trials.

The findings of the present review should be interpreted in light of key limitations. Firstly, of the 37 included trials, five were not explicitly labelled or described as hybrid type 1 effectiveness-implementation trial designs and were therefore classified as such based on whether they cited the original Curran et al. [1] publication and employed a methodology consistent with that of hybrid type 1 trials [28, 41, 50, 53, 74]. Therefore, it is possible that our search may have missed some hybrid type 1 trials that were not explicitly labelled as such. Our exclusion of non-English publications might bias the sample towards studies conducted in English-speaking countries. However, the exclusion of non-English publications has been shown not to significantly impact the direction or size of effect estimates [113]. Given most of the trials did not use Nilsen’s [4] taxonomy and there was heterogeneity between reporting in publications, classifying theoretical approaches used in hybrid type 1 RCTs proved challenging.

## Conclusions

This scoping review investigated the use of TMFs in hybrid type 1 effectiveness-implementation RCTs of healthcare interventions. It shows the majority of published hybrid type 1 RCTs report using at least one theoretical approach to explore the context for implementation of clinical interventions, with RE-AIM and CFIR being most common. Trials often lacked sufficient detail in reporting how TMFs were used. Future hybrid trials could address this gap by explicitly reporting whether and how TMFs are used.

## Supplementary Information

Additional file 1.

Additional file 2.

Additional file 3.

Additional file 4.

Additional file 5.

Additional file 6.

## Data Availability

All data generated or analysed during this review will be included in the published article or be available by request from the study team.

## Abbreviations

RCTs: Randomised control trials
TMFs: Theories, Models, Frameworks
