# Supplementary material for: The use of implementation science theoretical approaches in hybrid effectiveness-implementation type 1 randomised trials of healthcare interventions: A scoping review
Source: Implement Sci. 2025 May 16;20:23. doi: 10.1186/s13012-025-01435-6 (PMC12083051; doi:10.1186/s13012-025-01435-6)
Supplement: Supplementary file 2 — Additional file 2. [file 13012_2025_1435_MOESM2_ESM.docx]

**Additional file 2**

**Search Strategy**

The following tables present the search strategy used with the Medline (Ovid), EMBASE (Ovid), PsycINFO, CINAHL (EBSCO), and Cochrane CENTRAL.

**Medline (Ovid)**

| **Search** | **Query** | **Records retrieved from 31 October 2023** |
| --- | --- | --- |
| 1 | randomized controlled trial.pt. | 601,103 |
| 2 | controlled clinical trial.pt. | 95,404 |
| 3 | randomised.ab | 110,285 |
| 4 | placebo.ab | 220,867 |
| 5 | drug therapy.fs. | 2,631,097 |
| 6 | randomly.ab. | 352,263 |
| 7 | trial.ab. | 580,108 |
| 8 | groups.ab. | 2,188,317 |
| 9 | 1 or 2 or 3 or 4 or 5 or 6 or 7 or 8 | 5,183,208 |
| 10 | exp animals/ not humans.sh. | 5,163,555 |
| 11 | 9 not 10 | 4,458,200 |
| 12 | (hybrid adj3 (type* or stud* or design* or trial*)).ti.ab | 7,664 |
| 13 | 11 and 12 | 1,444 |
| 14 | Limit 13 to yr = “2012 – Current” | 1,195 |

| **Search** | **Query** | **Records retrieved from 26 November 2024** |
| --- | --- | --- |
| 1 | randomized controlled trial.pt. | 626,686 |
| 2 | controlled clinical trial.pt. | 95,622 |
| 3 | randomised.ab | 117,646 |
| 4 | placebo.ab | 229,675 |
| 5 | drug therapy.fs. | 2,758,710 |
| 6 | randomly.ab. | 372,202 |
| 7 | trial.ab. | 623,818 |
| 8 | groups.ab. | 2,316,861 |
| 9 | 1 or 2 or 3 or 4 or 5 or 6 or 7 or 8 | 5,456,152 |
| 10 | exp animals/ not humans.sh. | 5,282,378 |
| 11 | 9 not 10 | 4,701,085 |
| 12 | (hybrid adj3 (type* or stud* or design* or trial*)).ti.ab | 8,461 |
| 13 | 11 and 12 | 1,778 |
| 14 | Limit 13 to dt=20231031-20241126 [October 31^st^, 2023, November 26^th^, 2024] | 288 |

Search strategy based on Higgins JPT, Thomas J, Chandler J, Cumpston M, Li T, Page MJ, Welch VA (editors). *Cochrane Handbook for Systematic Reviews of Interventions* version 6.4 (updated August 2023). Cochrane, 2023. Available from www.training.cochrane.org/handbook.

**Embase (Ovid)**

| **Search** | **Query** | **Records retrieved from 31 October 2023** |
| --- | --- | --- |
| 1 | exp randomized controlled trial/ | 790,114 |
| 2 | Controlled clinical trial/ | 471,264 |
| 3 | random$.ti,ab. | 1,986,812 |
| 4 | randomization/ | 98,729 |
| 5 | intermethod comparison/ | 301,963 |
| 6 | placebo.ti,ab. | 366,855 |
| 7 | (compare or compared or comparison).ti. | 607,832 |
| 8 | ((evaluated or evaluate or evaluating or assessed or assess) and (compare or compared or comparing or comparison)).ab. | 2,796,356 |
| 9 | (open adj label).ti,ab. | 109,648 |
| 10 | ((double or single or doubly or singly) adj (blind or blinded or blindly)).ti,ab. | 275,053 |
| 11 | double blind procedure/ | 211,696 |
| 12 | parallel group$1.ti,ab. | 32,315 |
| 13 | (crossover or cross over).ti,ab. | 125,056 |
| 14 | ((assign$ or match or matched or allocation) adj5 (alternate or group$1 or intervention$1 or patient$1 or subject$1 or participant$1)).ti,ab. | 417,472 |
| 15 | (assigned or allocated).ti,ab. | 493,305 |
| 16 | (controlled adj7 (study or design or trial)).ti,ab. | 453,239 |
| 17 | (volunteer or volunteers).ti,ab. | 283,437 |
| 18 | human experiment/ | 647,430 |
| 19 | trial.ti. | 404,399 |
| 20 | or/1-19 | 6,362,950 |
| 21 | (random$ adj sampl$ adj7 ("cross section$" or questionnaire$1 or survey$ or database$1)).ti,ab. not (comparative study/ or controlled study/ or randomi?ed controlled.ti,ab. or randomly assigned.ti,ab.) | 9,634 |
| 22 | Cross-sectional study/ not (exp randomized controlled trial/ or controlled clinical study/ or controlled study/ or randomi?ed controlled.ti,ab. or control group$1.ti,ab.) | 364,014 |
| 23 | (((case adj control$) and random$) not randomi?ed controlled).ti,ab. | 21,665 |
| 24 | Systematic review.ti,ab. not (trial or study).ti. | 329,252 |
| 25 | (nonrandom$ not random$).ti,ab. | 18,996 |
| 26 | "random field$".ti,ab. | 2,972 |
| 27 | (random cluster adj3 sampl$).ti,ab. | 1,592 |
| 28 | (review.ab. and [review.pt](http://review.pt).) not trial.ti. | 1,138,697 |
| 29 | "we searched".ab. and (review.ti. or [review.pt](http://review.pt).) | 49,781 |
| 30 | "update review".ab. | 136 |
| 31 | (databases adj4 searched).ab. | 63,205 |
| 32 | (rat or rats or mouse or mice or swine or porcine or murine or sheep or lambs or pigs or piglets or rabbit or rabbits or cat or cats or dog or dogs or cattle or bovine or monkey or monkeys or trout or marmoset$1).ti. and animal experiment/ | 1,225,060 |
| 33 | Animal experiment/ not (human experiment/ or human/) | 2,573,897 |
| 34 | or/21-33 | 4,404,673 |
| 35 | 20 not 34 | 5,598,266 |
| 36 | (hybrid adj3 (type* or stud* or design* or trial*)).ti,ab. | 11,681 |
| 37 | 35 and 36 | 2,325 |
| 38 | limit 37 to yr="2012 -Current" | 1,899 |

| **Search** | **Query** | **Records retrieved from 26 November 2024** |
| --- | --- | --- |
| 1 | exp randomized controlled trial/ | 856,059 |
| 2 | Controlled clinical trial/ | 474,409 |
| 3 | random$.ti,ab. | 2,144,161 |
| 4 | randomization/ | 100,374 |
| 5 | intermethod comparison/ | 309,622 |
| 6 | placebo.ti,ab. | 386,212 |
| 7 | (compare or compared or comparison).ti. | 639,491 |
| 8 | ((evaluated or evaluate or evaluating or assessed or assess) and (compare or compared or comparing or comparison)).ab. | 3,033,161 |
| 9 | (open adj label).ti,ab. | 120,436 |
| 10 | ((double or single or doubly or singly) adj (blind or blinded or blindly)).ti,ab. | 289,291 |
| 11 | double blind procedure/ | 225,916 |
| 12 | parallel group$1.ti,ab. | 34,659 |
| 13 | (crossover or cross over).ti,ab. | 131,509 |
| 14 | ((assign$ or match or matched or allocation) adj5 (alternate or group$1 or intervention$1 or patient$1 or subject$1 or participant$1)).ti,ab. | 446,974 |
| 15 | (assigned or allocated).ti,ab. | 528,928 |
| 16 | (controlled adj7 (study or design or trial)).ti,ab. | 489,361 |
| 17 | (volunteer or volunteers).ti,ab. | 294,082 |
| 18 | human experiment/ | 675,792 |
| 19 | trial.ti. | 443,133 |
| 20 | or/1-19 | 6,806,970 |
| 21 | (random$ adj sampl$ adj7 ("cross section$" or questionnaire$1 or survey$ or database$1)).ti,ab. not (comparative study/ or controlled study/ or randomi?ed controlled.ti,ab. or randomly assigned.ti,ab.) | 10,286 |
| 22 | Cross-sectional study/ not (exp randomized controlled trial/ or controlled clinical study/ or controlled study/ or randomi?ed controlled.ti,ab. or control group$1.ti,ab.) | 418,441 |
| 23 | (((case adj control$) and random$) not randomi?ed controlled).ti,ab. | 23,102 |
| 24 | Systematic review.ti,ab. not (trial or study).ti. | 380,538 |
| 25 | (nonrandom$ not random$).ti,ab. | 19,969 |
| 26 | "random field$".ti,ab. | 3,138 |
| 27 | (random cluster adj3 sampl$).ti,ab. | 1,699 |
| 28 | (review.ab. and [review.pt](http://review.pt).) not trial.ti. | 1,249,881 |
| 29 | "we searched".ab. and (review.ti. or [review.pt](http://review.pt).) | 55,796 |
| 30 | "update review".ab. | 147 |
| 31 | (databases adj4 searched).ab. | 72,811 |
| 32 | (rat or rats or mouse or mice or swine or porcine or murine or sheep or lambs or pigs or piglets or rabbit or rabbits or cat or cats or dog or dogs or cattle or bovine or monkey or monkeys or trout or marmoset$1).ti. and animal experiment/ | 1,275,917 |
| 33 | Animal experiment/ not (human experiment/ or human/) | 2,685,603 |
| 34 | or/21-33 | 4,727,150 |
| 35 | 20 not 34 | 5,969,924 |
| 36 | (hybrid adj3 (type* or stud* or design* or trial*)).ti,ab. | 13,094 |
| 37 | 35 and 36 | 2,781 |
| 38 | limit 37 to dd=20231031-20241126 [October 31st, 2023, November 26th, 2024] | 177 |

Search strategy based on ISSG Search Filters Resource – EMBASE RCT filter [Internet]. Sites.google.com. Available from: https://sites.google.com/a/york.ac.uk/issg-search-filters-resource/home/rcts/embase-rct-filter

**PsycINFO**

| **Search** | **Query** | **Records retrieved from 31 October 2023** |
| --- | --- | --- |
| 1 | random*.tw. | 244,379 |
| 2 | (hybrid adj3 (type* or stud* or design* or trial*)).ti.ab | 1,020 |
| 3 | 1 and 2 | 115 |

| **Search** | **Query** | **Records retrieved from 26 November 2024** |
| --- | --- | --- |
| 1 | random*.tw. | 260,561 |
| 2 | (hybrid adj3 (type* or stud* or design* or trial*)).ti.ab | 1,181 |
| 3 | 1 and 2 | 159 |
| 4 | limit 3 to up=20231031-20241126 | 40 |

Search strategy based on Eady AM, Wilczynski NL, Haynes RB. PsycINFO search strategies identified methodologically sound therapy studies and review articles for use by clinicians and researchers. J Clin Epidemiol. 2008;61(1):34-40.

**CINAHL (EBSCO)**

| **Search** | **Query** | **Search Models** | **Records retrieved from 31 October 2023** |
| --- | --- | --- | --- |
| 1 | MH Randomized controlled trials | Boolean/Phrase | 140,477 |
| 2 | MH double-blind studies | Boolean/Phrase | 54,563 |
| 3 | MH single-blind studies | Boolean/Phrase | 16,095 |
| 4 | MH random assignment | Boolean/Phrase | 81,794 |
| 5 | MH pretest-posttest design | Boolean/Phrase | 54,025 |
| 6 | MH cluster sample | Boolean/Phrase | 5,310 |
| 7 | TI (randomised OR randomized) | Boolean/Phrase | 144,697 |
| 8 | AB (random*) | Boolean/Phrase | 402,875 |
| 9 | TI (trial) | Boolean/Phrase | 186,077 |
| 10 | MH (sample size) AND AB (assigned OR allocated OR control) | Boolean/Phrase | 4,442 |
| 11 | MH (placebos) | Boolean/Phrase | 14,230 |
| 12 | PT (randomized controlled trial) | Boolean/Phrase | 154,115 |
| 13 | AB (control W5 group) | Boolean/Phrase | 146,584 |
| 14 | MH (crossover design) OR MH (comparative studies) | Boolean/Phrase | 487,521 |
| 15 | AB (cluster W3 RCT) | Boolean/Phrase | 501 |
| 16 | MH animals+ | Boolean/Phrase | 105,187 |
| 17 | MH (animal studies) | Boolean/Phrase | 154,664 |
| 18 | TI (animal model*) | Boolean/Phrase | 3,874 |
| 19 | S16 or S17 or S18 | Boolean/Phrase | 250,929 |
| 20 | MH (human) | Boolean/Phrase | 2,731,982 |
| 21 | S19 NOT S20 | Boolean/Phrase | 216,606 |
| 22 | S1 OR S2 OR S3 OR S4 OR S5 OR S6 OR S7 OR S8 OR S9 OR S10 OR S11 OR S12 OR S13 OR S14 OR S15 | Boolean/Phrase | 1,033,543 |
| 23 | S22 NOT S21 | Boolean/Phrase | 985,341 |
| 24 | TI ( (hybrid N3 (type* or stud* or design* or trial*) ) OR AB ( (hybrid N3 ( type* or stud* or design* or trial*) ) | Boolean/Phrase | 1,463 |
| 25 | S23 AND S24 | Boolean/Phrase  Limiters – Published Date: 20120101-20231231 | 773 |

| **Search** | **Query** | **Search Models** | **Records retrieved from 26 November2024** |
| --- | --- | --- | --- |
| 1 | MH Randomized controlled trials | Boolean/Phrase | 147,371 |
| 2 | MH double-blind studies | Boolean/Phrase | 54,527 |
| 3 | MH single-blind studies | Boolean/Phrase | 16,153 |
| 4 | MH random assignment | Boolean/Phrase | 88,301 |
| 5 | MH pretest-posttest design | Boolean/Phrase | 57,291 |
| 6 | MH cluster sample | Boolean/Phrase | 5,757 |
| 7 | TI (randomised OR randomized) | Boolean/Phrase | 155,799 |
| 8 | AB (random*) | Boolean/Phrase | 409,489 |
| 9 | TI (trial) | Boolean/Phrase | 200,525 |
| 10 | MH (sample size) AND AB (assigned OR allocated OR control) | Boolean/Phrase | 4,483 |
| 11 | MH (placebos) | Boolean/Phrase | 14,567 |
| 12 | PT (randomized controlled trial) | Boolean/Phrase | 159,868 |
| 13 | AB (control W5 group) | Boolean/Phrase | 151,711 |
| 14 | MH (crossover design) OR MH (comparative studies) | Boolean/Phrase | 508,290 |
| 15 | AB (cluster W3 RCT) | Boolean/Phrase | 518 |
| 16 | MH animals+ | Boolean/Phrase | 102,128 |
| 17 | MH (animal studies) | Boolean/Phrase | 154,239 |
| 18 | TI (animal model*) | Boolean/Phrase | 3,945 |
| 19 | S16 or S17 or S18 | Boolean/Phrase | 247,478 |
| 20 | MH (human) | Boolean/Phrase | 2,833,639 |
| 21 | S19 NOT S20 | Boolean/Phrase | 212,848 |
| 22 | S1 OR S2 OR S3 OR S4 OR S5 OR S6 OR S7 OR S8 OR S9 OR S10 OR S11 OR S12 OR S13 OR S14 OR S15 | Boolean/Phrase | 1,073,644 |
| 23 | S22 NOT S21 | Boolean/Phrase | 1,025,492 |
| 24 | TI ( (hybrid N3 (type* or stud* or design* or trial*) ) OR AB ( (hybrid N3 ( type* or stud* or design* or trial*) ) | Boolean/Phrase | 1,625 |
| 25 | S23 AND S24 | Boolean/Phrase  Limiters – Published Date: 20231031-20241126 | 100 |

Search strategy based on Glanville J, Dooley G, Wisniewski S, Foxlee R, Noel-Storr A. Development of a search filter to identify reports of controlled clinical trials within CINAHL Plus. *Health Information & Libraries Journal*. 2019;36:73-90

**Cochrane CENTRAL**

Record Title (hybrid NEAR/3 (type* or stud* or design* or trial*)) OR Abstract (hybrid NEAR/3 (type* or stud* or design* or trial*))

Results: 1424 records retrieved from 31 October 2023

Results: 467 records retrieved from search on 26 November 2024

**Scopus**

Citation tracking of “Effectiveness-implementation hybrid designs: Combining elements of clinical effectiveness and implementation research to enhance public health impact”

Results: 1,982 records retrieved from 31 October 2023

Citation tracking of “Effectiveness-implementation hybrid designs: Combining elements of clinical effectiveness and implementation research to enhance public health impact”

Results: 471 records retrieved from 26 November 2024
