# Supplementary material for: The use of implementation science theoretical approaches in hybrid effectiveness-implementation type 1 randomised trials of healthcare interventions: A scoping review
Source: Implement Sci. 2025 May 16;20:23. doi: 10.1186/s13012-025-01435-6 (PMC12083051; doi:10.1186/s13012-025-01435-6)
Supplement: Supplementary file 3 — Additional file 3. [file 13012_2025_1435_MOESM3_ESM.docx]

**Additional File 3**

**Data Extraction Form**

**Study characteristics:**

| **Item** | **Extracted data** |
| --- | --- |
| Title |  |
| Year |  |
| Lead author surname |  |
| Source of publication | e.g Journal name |
| Country where study conducted |  |
| Study objective |  |
| Study setting (e.g. hospital, primary care) |  |
| Intervention recipients | Age:  Gender:  Education level:  Ethnicity:  Other: |
| Clinical problem |  |
| Clinical intervention name |  |
| Clinical intervention: brief description |  |
| Implementation design (ie, to describe the context for implementation) | Mixed methods  Qualitative  Quantitative  Other (specify) ______ |
| Implementation outcomes |  |
| Implementation participants | Patients  Name type here _______  Providers  Name type here _______  Stakeholders:  Name type here ________  Other: specify _______ |
| Number of participants involved in the implementation component of the trial | Patient:  Providers:  Stakeholders:  Other: |

**Theories, Models and Frameworks**

****Fill in only the amount relevant for the paper**

| Does it us a theory, model, or framework? | Yes  No |
| --- | --- |
| Name the theory, model, or framework |  |
| Category of theory, model, or framework | Process model  Determinant framework  Classic theory  Implementation theory  Evaluation framework  Not sure |
| How was it used? | Informed by  Applied  Cited  Other use (specify) _______ |
| Notes (extent to which T,M,F was applied or used) |  |

| Does it us another theory, model, or framework? | Yes  No |
| --- | --- |
| Name the theory, model, or framework |  |
| Category of theory, model, or framework | Process model  Determinant framework  Classic theory  Implementation theory  Evaluation framework  Not sure |
| How was it used? | Informed by  Applied  Cited  Other use (specify) _______ |
| Notes (extent to which T,M,F was applied or used) |  |

| Does it us another theory, model, or framework? | Yes  No |
| --- | --- |
| Name the theory, model, or framework |  |
| Category of theory, model, or framework | Process model  Determinant framework  Classic theory  Implementation theory  Evaluation framework  Not sure |
| How was it used? | Informed by  Applied  Cited  Other use (specify) _______ |
| Notes (extent to which T,M,F was applied or used) |  |
