# Supplementary material for: The use of implementation science theoretical approaches in hybrid effectiveness-implementation type 1 randomised trials of healthcare interventions: A scoping review
Source: Implement Sci. 2025 May 16;20:23. doi: 10.1186/s13012-025-01435-6 (PMC12083051; doi:10.1186/s13012-025-01435-6)
Supplement: Supplementary file 4 — Additional file 4. [file 13012_2025_1435_MOESM4_ESM.docx]

|  | **Additional File 4**  **Studies requiring clarification** |  | | |
| --- | --- | --- | --- | --- |
| **References** | **Title** | **Question** | **Decision** | **Reasoning** |
| (1) | Adaptation of an Evidence-Based Diabetes Management Intervention for Delivery in Community Settings: Findings From a Pilot Randomized Effectiveness Trial | Is this a hybrid type 1 despite not explicitly mentioning it? | Include | Explores barriers and facilitators of potential implementation, therefore we are considering it a Hybrid type 1. |
| (2) | Addressing Diabetes in Primary Care: Hybrid Effectiveness-Implementation Study of Lifestyle Redesign R Occupational Therapy. | Is this a hybrid type 1 despite not explicitly mentioning it? | Exclude | Noted implementation outcomes, therefore we are considering it a Hybrid type 2. |
| (3) | Adolescents’ experiences with group antenatal care: Insights from a mixed-methods study in Senegal | Is this a hybrid type 1 despite not explicitly mentioning it? | Include | There is no explanation of an implementation strategy and it explores the best methods to **introduce intervention into practice,** therefore we are considering it a Hybrid type 1. |
| (4) | Association of Screening and Brief Intervention With Substance Use in Massachusetts Middle and High Schools | Does this fit every criteria of ours to be included? | Exclude | It does not state that it is randomised. |
| (5) | Barriers and facilitators to implementing a continuing medical education intervention in a primary health care setting | Is this a hybrid type 1 despite explicitly mentioning that is it a hybrid type 1? | Exclude | Information from the trial registration revealed that the intervention is an educational strategy targeting primary care providers with the aim of improving Benzodiazepine. Despite the authors labelling the study as a Hybrid type 1, based on the information from the trial registration, we are not considering it a Hybrid type 1. |
| (6) | Barriers and Facilitators to the Implementation of a Mobile Insulin Titration Intervention for Patients With Uncontrolled Diabetes: A Qualitative Analysis | Does this fit every criteria of ours to be included? | Exclude | It does not state that it is randomised. |
| (7) | Enhancing Reach Out and Read With a Video and Text Messages: A Randomized Trial in a Low-Income Predominantly Latino Sample | Does this intervention meet our inclusion criteria? | Exclude | It is not a healthcare intervention as it did not aim to improve health as shown by the outcome measures for the effectiveness trial, none were based on improving an aspect of the participant's health |
| (8) | Evaluating an implementation programme for medication review with follow-up in community pharmacy using a hybrid effectiveness study design: translating evidence into practice | Does this fit every criteria of ours to be included? | Exclude | It does not state that it is randomised. |
| (9) | Evaluating the Implementation of a Multicomponent Intervention Consisting of Education and Feedback on Reducing Benzodiazepine Prescriptions by General Practitioners: BENZORED Hybrid Type I Cluster Randomized Controlled Trial | Is this a hybrid type 1 despite explicitly mentioning that is it a hybrid type 1? | Exclude | Although it considers itself a Hybrid type 1, we are considering it as mislabelled as it is an implementation paper and looking at how to implement the intervention within practitioners rather than investigating facilitators and barriers. |
| (10) | Evaluating the implementation of a multidisciplinary lifestyle intervention for people with severe mental illness in sheltered housing: effectiveness-implementation hybrid randomised controlled trial | Does this intervention meet our inclusion criteria? | Include | It is considered a healthcare intervention as they have a mental illness and it is about improving their lifestyle. |
| (11) | Impact of the CARD (Comfort Ask Relax Distract) system on school-base-vaccinations: A cluster randomized trial | Does this fit every criteria of ours to be included? | Exclude | It is not the correct setting as it is conducted in a school. |
| (12) | Implementation and adoption of a health insurance support tool in the electronic health record: a mixed methods analysis within a randomized trial | Does this fit every criteria of ours to be included? | Exclude | It is not a healthcare intervention as it is based on health records rather than improving health. |
| (13) | Implementation and effectiveness of a multi‐domain program for older adults at risk of cognitive impairment at neighbourhood senior centres | Does this setting meet our inclusion criteria? | Exclude | One setting in the trial was a senior activity centre and another was a senior care centre, considering a senior activity centre is not the correct setting, we will consider it as not the correct setting. |
| (14) | Implementation of a complex intervention to improve hospital discharge: process evaluation of a cluster randomised controlled trial | Is this a hybrid type 1 despite not explicitly mentioning it? | Exclude | It described an education session about the intervention, and based on the description of the intervention, we are considering it a Hybrid type 2. |
| (15) | Implementation of a proactive referral tool for child healthcare professionals to encourage and facilitate parental smoking cessation in the Netherlands: a mixed- methods study | Is this a hybrid type 1 despite not explicitly mentioning it? | Exclude | Discusses implementing an implementation strategy, therefore we are considering it a Hybrid type 2. |
| (16) | Implementation of a Web-Based Communication System for Primary Care Providers and Cancer Specialists | Does this intervention meet our inclusion criteria? | Exclude | It is not a healthcare intervention as it is based on communication amongst primary care providers rather than improving health. |
| (17) | Integrating opioid use disorder treatment into primary care settings | Is this a hybrid type 1 despite not explicitly mentioning it? | Exclude | It says evaluating the implementation of the intervention, therefore we are considering it a Hybrid type 2. |
| (18) | A multifaceted provider-centred intervention versus usual care to improve the recognition and diagnosis of depression in primary health care: a hybrid study | Does this fit every criteria of ours to be included? | Exclude | It does not state that it is randomised. |
| (19) | A nurse-led intervention to reduce the incidence and duration of delirium among adults admitted to intensive care: A stepped-wedge cluster randomised trial" | Is this a hybrid type 1 despite not explicitly mentioning it? | Exclude | It mentioned that it assesses the dissemination of the intervention, therefore we are not considering it a Hybrid type 1 |
| (20) | Patient and Healthcare Provider Perspectives on the Implementation of a Web-Based Clinical Communication System for Cancer: A Qualitative Study | Is this a hybrid type 1 despite not explicitly mentioning it? | Exclude | It is not a healthcare intervention as it is based on communication amongst primary care providers rather than improving health. |
| (21) | Scaling up Action Schools! BC: How Does Voltage Drop at Scale Affect Student Level Outcomes? A Cluster Randomized Controlled Trial | Does this setting meet our inclusion criteria? | Exclude | It is not the correct setting as it is conducted in a school. |
| (22) | The Use of Dissemination and Implementation to Improve Multimodal Analgesia in Head and Neck Surgery | Is this a hybrid type 1 despite not explicitly mentioning it? | Exclude | It does not speak about implementation. |
| (23) | User experiences of an app-based mHealth intervention (MINISTOP 2.0) integrated in Swedish primary child healthcare among Swedish-, Somali- and Arabic-speaking parents and child healthcare nurses: A qualitative study | Does this intervention meet our inclusion criteria? | Exclude | It is not a healthcare intervention as it is health promotion rather than health prevention. |
| References:  1. Ellis DA, Carcone AI, Naar-King S, Rajkumar D, Palmisano G, Moltz K. Adaptation of an Evidence-Based Diabetes Management Intervention for Delivery in Community Settings: Findings From a Pilot Randomized Effectiveness Trial. J Pediatr Psychol. 2019;44(1):110-25.  2. Pyatak E, King M, Vigen CLP, Salazar E, Diaz J, Schepens Niemiec SL, et al. Addressing Diabetes in Primary Care: Hybrid Effectiveness-Implementation Study of Lifestyle Redesign R Occupational Therapy. The American journal of occupational therapy : official publication of the American Occupational Therapy Association. 2019;73(5):7305185020p1-p12.  3. Vandermorris A, McKinnon B, Sall M, Witol A, Traore M, Lamesse-Diedhiou F, et al. Adolescents' experiences with group antenatal care: Insights from a mixed-methods study in Senegal. Tropical Medicine and International Health. 2021;26(12):1700-8.  4. Levy S, Wisk LE, Minegishi M, Ertman B, Lunstead J, Brogna M, et al. Association of Screening and Brief Intervention With Substance Use in Massachusetts Middle and High Schools. JAMA network open. 2022;5(8):e2226886.  5. Reis T, Faria I, Serra H, Xavier M. Barriers and facilitators to implementing a continuing medical education intervention in a primary health care setting. BMC health services research. 2022;22(1):638.  6. Rogers E, Aidasani SR, Friedes R, Hu L, Langford AT, Moloney DN, et al. Barriers and facilitators to the implementation of a mobile insulin titration intervention for patients with uncontrolled diabetes: A qualitative analysis. JMIR mHealth uHealth. 2019;7(7).  7. Jimenez ME, Crabtree BF, Hudson SV, Mendelsohn AL, Lima D, Shelton PA, et al. Enhancing Reach Out and Read With a Video and Text Messages: A Randomized Trial in a Low-Income Predominantly Latino Sample. Academic pediatrics. 2021;21(6):968-76.  8. Varas-Doval R, Gastelurrutia MA, Benrimoj SI, Zarzuelo MJ, Garcia-Cardenas V, Perez-Escamilla B, et al. Evaluating an implementation programme for medication review with follow-up in community pharmacy using a hybrid effectiveness study design: translating evidence into practice. BMJ open. 2020;10(9):e036669.  9. Socias I, Leiva A, Pombo-Ramos H, Bejarano F, Sempere-Verdu E, Rodriguez-Rincon RM, et al. Evaluating the Implementation of a Multicomponent Intervention Consisting of Education and Feedback on Reducing Benzodiazepine Prescriptions by General Practitioners: BENZORED Hybrid Type I Cluster Randomized Controlled Trial. International journal of environmental research and public health. 2021;18(15).  10. Smit MMC, Waal ED, Tenback DE, Deenik J. Evaluating the implementation of a multidisciplinary lifestyle intervention for people with severe mental illness in sheltered housing: effectiveness-implementation hybrid randomised controlled trial. BJPsych Open. 2022;8(6):e201.  11. Taddio A, Gudzak V, Jantzi M, Logeman C, Bucci LM, MacDonald NE, et al. Impact of the CARD (Comfort Ask Relax Distract) system on school-based vaccinations: A cluster randomized trial. Vaccine. 2022;40(19):2802-9.  12. Hatch B, Tillotson C, Huguet N, Marino M, Baron A, Nelson J, et al. Implementation and adoption of a health insurance support tool in the electronic health record: a mixed methods analysis within a randomized trial. BMC health services research. 2020;20(1):428.  13. Ng PEM, Nicholas SO, Wee SL, Yau TY, Chan A, Chng I, et al. Implementation and effectiveness of a multi-domain program for older adults at risk of cognitive impairment at neighborhood senior centres. Sci Rep. 2021;11(1).  14. Rachamin Y, Grischott T, Neuner-Jehle S. Implementation of a complex intervention to improve hospital discharge: process evaluation of a cluster randomised controlled trial. BMJ open. 2021;11(5):e049872.  15. Scheffers-van Schayck T, Hipple Walters B, Otten R, Kleinjan M. Implementation of a proactive referral tool for child healthcare professionals to encourage and facilitate parental smoking cessation in the Netherlands: a mixed-methods study. BMC Health Serv Res. 2021;21(1).  16. Petrovic B, Bender JL, Liddy C, Afkham A, McGee SF, Morgan SC, et al. Implementation of a Web-Based Communication System for Primary Care Providers and Cancer Specialists. Curr Oncol. 2023;30(3):3537-48.  17. Austin EJ, Chen J, Briggs ES, Ferro L, Barry P, Heald A, et al. Integrating Opioid Use Disorder Treatment Into Primary Care Settings. JAMA network open. 2023;6(8):e2328627.  18. Nogueras EV, Cantero N, Macias M, Morales-Asencio JM, Garcia-Herrera Perez-Bryan JM, Hurtado MM. A multifaceted provider-centred intervention versus usual care to improve the recognition and diagnosis of depression in primary health care: a hybrid study. Primary health care research & development. 2023;24(100897390):e45.  19. Brennan K, Sanchez D, Hedges S, Lynch J, Hou YC, Al Sayfe M, et al. A nurse-led intervention to reduce the incidence and duration of delirium among adults admitted to intensive care: A stepped-wedge cluster randomised trial. Aust Crit Care. 2023;36(4):441-8.  20. Petrovic B, O’Brien MA, Liddy C, Afkham A, McGee SF, Morgan SC, et al. Patient and Healthcare Provider Perspectives on the Implementation of a Web-Based Clinical Communication System for Cancer: A Qualitative Study. Curr Oncol. 2022;29(11):8401-14.  21. Nettlefold L, Naylor PJ, Macdonald HM, McKay HA. Scaling up action schools! bc: How does voltage drop at scale affect student level outcomes? a cluster randomized controlled trial. Int J Environ Res Public Health. 2021;18(10).  22. Shnayder Y, Baumanis MM, Brown A, Reese A, Bur AM, Kakarala K, et al. The Use of Dissemination and Implementation to Improve Multimodal Analgesia in Head and Neck Surgery. Laryngoscope. 2023;133(S5):S1-S11.  23. Alexandrou C, Rutberg S, Johansson L, Lindqvist AK, Müssener U, Löf M. User experiences of an app-based mHealth intervention (MINISTOP 2.0) integrated in Swedish primary child healthcare among Swedish-, Somali- and Arabic-speaking parents and child healthcare nurses: A qualitative study. Digit Health. 2023;9. | | | | |
