# Supplementary material for: The use of implementation science theoretical approaches in hybrid effectiveness-implementation type 1 randomised trials of healthcare interventions: A scoping review
Source: Implement Sci. 2025 May 16;20:23. doi: 10.1186/s13012-025-01435-6 (PMC12083051; doi:10.1186/s13012-025-01435-6)
Supplement: Supplementary file 5 — Additional file 5. [file 13012_2025_1435_MOESM5_ESM.docx]

**Additional file 5:**

**Hybrid type 1 RCT characteristics**

|  | No. of trials (*%*) |
| --- | --- |
| **Geographic region** |  |
| North America | 25 (*67.6*) |
| Africa | 5 (*13.5*) |
| Europe | 4 (*10.8*) |
| Australia and New Zealand | 2 (*5.4)* |
| Central and South America | 1 (*2.7*) |
| **Trial setting** |  |
| Community care | 17 (*45.9*) |
| Primary care | 9 (*24.3*) |
| Veterans’ affairs settings | 7 (18*.9*) |
| Hospitals | 2 (*5.4*) |
| Nursing homes and aged care settings | 2 (*5.4*) |
| **Participants in effectiveness component of trial** |  |
| Adults | 16 (*43.2*) |
| Not specified | 6 (*16.2*) |
| Women only | 4 (*10.8*) |
| Veterans | 4 (*10.8*) |
| Children | 3 (*8.1*) |
| Older adults (65 years and older) | 3 (*8.1*) |
| Male only | 1 (*2.7*) |
| **Clinical problem targeted by intervention** |  |
| Mental health and well-being | 12 (*32.4*) |
| Substance use (inc. tobacco) | 6 (*16.2*) |
| Diabetes | 5 (*13.5*) |
| Falling risk in older adults | 3 (*8.1*) |
| Cancer | 2 (*5.4*) |
| HIV/AIDS | 2 (*5.4*) |
| Pregnancy-related | 2 (*5.4*) |
| Diseases (non-HIV) | 2 (*5.4*) |
| Dementia | 1 (*2.7*) |
| Heart failure | 1 (*2.7*) |
| Pain | 1 (*2.7*) |
| **Data collection method** |  |
| Interviews and questionnaires or checklists | 10 (*27.0*) |
| Interviews only | 9 (*24.3*) |
| Questionnaires or checklists only | 4 (*10.8*) |
| Chart reviews/report reviews | 3 (*8.1*) |
| Questionnaires or checklists, interviews and/or focus groups | 3 (*8.1*) |
| Interviews and focus groups | 2 (*5.4*) |
| Chart review, cost tracking analysis and questionnaires or checklists and interviews | 1 (*2.7*) |
| Cost tracking analysis | 1 (*2.7*) |
| Duration and content of implementation, uptake of the intervention and its impact on clinical management, interviews and focus groups | 1 (*2.7*) |
| Focus groups only | 1 (*2.7*) |
| Focus groups and questionnaires or checklists | 1 (*2.7*) |
| Interviews and meetings | 1 (*2.7*) |
| **Implementation participants** |  |
| Intervention providers | 10 (*28.6*) |
| Participants receiving the intervention and intervention providers | 9 (*25.7*) |
| Participants receiving the intervention | 6 (*17.1*) |
| Stakeholders | 4 (*11.4*) |
| Participants receiving the intervention, intervention providers, and stakeholders | 2 (*5.7*) |
| Participants receiving the intervention and stakeholders | 3 (*8.6*) |
| Intervention providers and stakeholders | 1 (*2.9*) |

*Please note that this table only includes the data collection methods used in the implementation publications from our review. It does not list all methods mentioned in the trial protocols if the corresponding implementation outcome publications were not found. Additionally, two trials did not have implementation participants as they focussed on cost analysis and the number of sessions attended per participant. As a result, there are 35 trials in total in the implementation participants section of the table.*

**Trial characteristics**

| **Intervention names (trial citation)** | **Year** | **Participants** | **Setting** | **Clinical problem** | **Implementation constructs** |
| --- | --- | --- | --- | --- | --- |
| Acceptance and commitment therapy (ACT) ^(1)^ | 2016 | Veteran health administration inpatients with current or recent psychotic symptoms diagnosed with psychotic or mood disorder | Veterans’ health administration inpatient setting | Psychosis | Feasibility, acceptability, safety, barriers and facilitators to patient participation and implementation of ACT |
| Activity Tracker (Fitbit Charge HR, Fitbit Inc.) ^(2)^ | 2023 | Adults with type 2 diabetes | Primary care settings | Type 2 diabetes | Acceptability, satisfaction of the portable monitoring device and compliance, perceptions of implementation in a primary care setting |
| AI-Anon Intensive Referral (AIR) ^(3)^ | 2022 | Not specified | Veterans’ affairs and substance abuse disorders clinics | Substance use disorders | Intervention characteristics, outer setting, inner setting, staff characteristics, process |
| An Individualised Mental Health Intervention for Autism Spectrum Disorder (AIM HI) ^(4, 5)^ | 2019-2020 | Children who had an existing autism spectrum disorder diagnosis | Community mental health settings | Mental health in autistic children | Fit, acceptability and sustained implementation of the intervention and classify the content, nature, and goals of the adaptations for a specific subgroup |
| Assertive community treatment + cognitive behavioural social skills training ^(6, 7)^ | 2015-2019 | Adults with a diagnosis of schizophrenia or schizoaffective disorder | Community based organisations | Schizophrenia or schizoaffective disorder | Barriers and facilitators to implementation using concept mapping – characterising and assessing the implementation experience from multiple stakeholder perspectives to inform future implementation initiatives |
| Cancer SurvivorLink ^(8)^ | 2023 | Not specified, can infer from setting that it is children | Survivorship clinics | Paediatric cancer | Perceptions of participants knowledge about intervention, facilitators and barriers related to engagement in intervention at all levels of the socio-ecological model framework: Individual and intrapersonal, organisational factors, community factors, societal/policy factors |
| Collaborative care package ^(9, 10)^ | 2022 | Not specified | Primary healthcare clinics | Depression comorbid with physical conditions | Reach, adoption and implementation |
| Cradle Vital Signal Alert ^(11)^ | 2019 | Women identified as pregnant or within 42 days of delivery, that presented to routine maternity care | Maternity care in low resource settings | Maternal mortality | Implementation fidelity, reach and adoption |
| Diabetes Prevention Program ^(12, 13)^ | 2020-2021 | Adults with prediabetes, defined by 5,7% ≤ HbA1c ≤ 6.4 | Primary care | Diabetes | Adoption: number, proportion and representativeness of participating physicians/providers, value and practicality of adopting program within their facility  Implementation: degree to which intervention is delivered as intended, participant engagement, participant perceptions of program, provider and administrator perceptions of program evidence, clinical culture, and facilitation for future adoption, implementation, and sustainability |
| Electronic family health history collection tool and decision support platform (MeTree) ^(14, 15)^ | 2023 | 40-64 years old with no evidence of increased colorectal cancer risk | Healthcare system and hospital | Colorectal cancer | Economic analysis and experience with clinical decision support reports and effects on primary care provider workflow |
| Empowering patients in chronic care (EPICC) ^(16)^ | 2022 | Adults with diabetes | Veterans’ affairs clinic | Diabetes-associated distress and Hemoglobin A_1c_ levels | Reach, adoption, implementation (number of sessions attended per patient) |
| Group Antenatal Care (G-ANC) ^(17)^ | 2021 | Female adolescents | Antenatal care | Prenatal health in teens | Coverage and acceptability |
| Integrated Coaching for Better Mood and Weight (I-CARE) ^(18-20)^ | 2015-2021 | Adults with clinically significant depression | Primary care setting | Weight and depression | Reach (features of I-CARE that enhanced reach and how it may be better achieved), Adoption (stakeholders perspectives on what they might recommend)  Implementation (participants report of I-CARE strategies, stakeholder roles in implementing I-Care and stakeholders’ recommendations for improvement) Maintenance (stakeholders perceptions of how to sustain and maintain the I-CARE program) |
| Incentive intervention for substance use disorders ^(21)^ | 2014 | Veterans | Veterans’ health administration medical centres | Alcohol dependence and/or stimulant dependence | Reach, effectiveness, adoption, implementation, maintenance, evidence and context (from PARIHS) |
| INTEGRA- Medication for opioid use disorder and HIV care ^(22)^ | 2024 | Adults who inject drugs with human papillomavirus | Community setting | Substance use | Facilitator/barriers to INTEGRA, Reach, Adoption, Implementation, and potential Maintenance; and associations with clinical effectiveness outcomes |
| Interpersonal psychotherapy ^(23, 24)^ | 2016-2021 | Human immunodeficiency virus infection (HIV) positive women | HIV clinic | Depression and post traumatic stress disorder | Cost-benefit, acceptability, appropriateness, feasibility and fidelity |
| The Khanya Intervention ^(25-27)^ | 2020-2022 | Individuals with Human Papillomavirus | On-site alcohol and other drug treatment centre | Alcohol and other drug use | Feasibility, acceptability, fidelity, and barriers and facilitators to implementation |
| Modular Anxiety Skills Training (MAST-V) ^(28)^ | 2024 | Veterans | Primary care | Anxiety | Treatment fidelity, barriers and facilitators to implementation |
| mHealth intervention ^(29-32)^ | 2019-2023 | Women who performed human papillomavirus self-collection during community health workers home visit and was able to provide a mobile phone number | Community healthcare home visits | Human Papillomavirus | Reach: representativeness of women reached by the intervention, Effectiveness: in increasing womens’ adherence to triage, Adoption: by community health workers of the strategy or visiting HPV+ women after receiving SMS messages and e-mails,  Acceptability: of the intervention by adopters, Implementation: of intervention activities according to protocol, Acceptability: of the intervention by women, barriers and facilitators to implementing and administering the intervention, Maintenance |
| MULTidisciplinary lifestyle-enhancing support for patients living in Sheltered Housing (MULTI-SH) ^(33)^ | 2022 | Adults with schizophrenia spectrum, major depressive or bipolar disorder living in sheltered housing facilities | Housing facilities | Lifestyle behaviours in those with severe mental illness | Adoption, acceptability, feasibility, and fidelity |
| MyFood ^(34)^ | 2021 | Adults | Hospital | Disease-related malnutrition | Reach: proportion of patients who participated in the trial and reasons for decline (register of participants), Adoption: the proportion of nurses who used the MyFood system (log data, nurse questionnaires), Implementation: fidelity to the MyFood system. attitudes and experiences with the use. Usability of MyFood (questionnaires to nurses, focus groups with nurse, interview with participants)  Implementation: the extent to which the intervention was sustained over time. *Note: Data collection was conducted over a limited period; hence they did not have the opportunity to study maintenance over a long period. |
| Peer-led group lifestyle balance (PGLB) ^(35-38)^ | 2019-2013 | Adults with co-occurring disorders (i.e. serious mental illness and substance use disorder) | Supportive housing agencies | Serious mental illness | System-level and organisational-level factors, staff-level factors |
| Pharmacogenetic (PGX) test ^(39)^ | 2020 | Not specified | Primary care | Major depressive disorder | Consolidated Framework for Implementation Research (CIFR) domain constructs; Evidence, relative advantage, adaptability, trialability, complexity, and intervention design |
| Positive Health Check ^(40-42)^ | 2022 | Adults with Human Immunodeficiency virus diagnosis | Primary care and mental health outpatient clinics | Human Immunodeficiency virus | Innovation values fit, organisational readiness for change, implementation climate |
| Rapid Education/Encouragement and Communications for Health (REACH) ^(43, 44)^ | 2018-2022 | Adults with diagnosis of Type 2 Diabetes | Safety net clinics | Type 2 Diabetes | Inner setting, outer setting, implementation process, characteristics of the individuals, and characteristics of the intervention |
| Reach for Control (RFC) ^(45)^ | 2019 | Children with type 1 diabetes | Community setting | Diabetes | Intervention feasibility and acceptability |
| Rehabilitation EnAblement in Chronic Heart Failure (REACH-HF) ^(46)^ | 2022 | People with heart failure | Home-based intervention | Heart Failure | Behaviour change, contextual factors, experience of living with disease, fidelity |
| Relation Agent for alcohol brief intervention ^(47, 48)^ | 2023 | Veterans who received services through the Veteran Affairs primary care clinic | Veterans’ affairs primary care clinics | Unhealthy drinking | Appearance, setting and preferences |
| SMART program ^(49)^ | 2020 | Male Adolescents | Community based organisations, including health centres | Unsafe practice (Human Immunodeficiency Virus Infection prevention) | Reach, effectiveness or efficacy, adoption, implementation, sustainability.  CFIR: Organisation level measures: inner setting and characteristics of community-based organisations as well as reach and engage adolescent men who have sex with men |
| Stay Up Right ^(50)^ | 2023 | Older adults | Long term care facility | Falling risk | Factors influencing the maintenance of Stay Up Right as usual practice |
| Strategies to Reduce Injuries and Develop confidence in Elders (STRIDE) ^(51)^ | 2021 | Older adults | Clinical trial sites | Fall related injury | Intervention characteristics, outer setting, inner setting, characteristics of individuals who receive, deliver, or facilitate the intervention, process |
| The Family Check up (FCU) ^(52)^ | 2015 | Primary caregivers and children | Community mental health agencies | Paediatric mental health | Adoption, acceptability, feasibility, and fidelity |
| The Smoking Treatment for Ontario Patients (STOP) + Clinical decision support systems (CDSSs) ^(53, 54)^ | 2020- 2022 | Treatment-seeking cigarette smokers who reported physical activity levels and fruit and vegetable consumptions levels that were lower than the national guidelines | Primary care practice | Tobacco use | Reach, effectiveness, adoption, implementation and maintenance |
| TOP UP ^(55, 56)^ | 2023 | Older people receiving aged care services | Community setting | Fall related injury | Acceptability, reach fidelity, dose delivered, and adoption |
| Present Centered Therapy and Mantram Repetition Program ^(57)^ | 2020 | Veteran patients | Veterans’ affairs medical centre | Post traumatic stress | Self-reported referral behaviour, provider's views on opportunities to learn about different clinical practices, who they speak to when they want to learn more information on these practices, and what is most helpful to them to make this learning possible |
| Whole Health Team ^(58)^ | 2020 | Not specified | Veterans’ affairs clinical settings | Chronic Pain | Evidence (scientific rationale for intervention implementation), context (various local contextual factors likely to affect implementation), facilitation (strategies approaches to guiding/supporting local implementation), assess the feasibility and acceptability of the study interventions, identify potential facilitators and barriers to implementing and staffing each clinical study arm and recruiting and enrolling veterans into the study |
| Wittener model of case conferences for people with dementia – the Innovative dementia-oriented Assessment tool (WELCOME-IdA) ^(59, 60)^ | 2014-2021 | Not specified | Nursing homes | Dementia | Intervention characteristics: relative advantage, adaptability, complexity, inner setting: tension for change, relative priority, learning climate, leadership engagement, process: opinion leaders, formally appointed internal implementation leaders, innovation participants and executing |

1. Boden MT, Gaudiano BA, Walser RD, Timko C, Faustman W, Yasmin S, et al. Feasibility and challenges of inpatient psychotherapy for psychosis: lessons learned from a veterans health administration pilot randomized controlled trial. BMC research notes. 2016;9(101462768):376.

2. Pelletier C, Chabot C, Gagnon MP, Rhéaume C. Implementing an Activity Tracker to Increase Motivation for Physical Activity in Patients With Diabetes in Primary Care: Strengths, Weaknesses, Opportunities and Threats (SWOT) Analysis. JMIR Form Res. 2023;7.

3. Baloh J, Curran GM, Timko C, Grant KM, Cucciare MA. Al-Anon Intensive Referral (AIR): A qualitative formative evaluation for implementation. J Subst Abuse Treat. 2022;132.

4. Brookman-Frazee L, Roesch S, Chlebowski C, Baker-Ericzen M, Ganger W. Effectiveness of training therapists to deliver an individualized mental health intervention for children with asd in publicly funded mental health services: A cluster randomized clinical trial. JAMA Psychiatry. 2019;76(6):574-83.

5. Chlebowski C, Hurwich-Reiss E, Wright B, Brookman-Frazee L. Using stakeholder perspectives to guide systematic adaptation of an autism mental health intervention for Latinx families: A qualitative study. Journal of community psychology. 2020;48(4):1194-214.

6. Granholm E, Holden JL, Sommerfeld D, Rufener C, Perivoliotis D, Mueser K, et al. Enhancing assertive community treatment with cognitive behavioral social skills training for schizophrenia: study protocol for a randomized controlled trial. Trials. 2015;16:438.

7. Sommerfeld DH, Aarons GA, Naqvi JB, Holden J, Perivoliotis D, Mueser KT, et al. Stakeholder Perspectives on Implementing Cognitive Behavioral Social Skills Training on Assertive Community Treatment Teams. Adm Policy Ment Health Ment Health Serv Res. 2019;46(2):188-99.

8. Krauss V, Mertens A, Marchak JG, Haardorfer R, Meacham LR, Lewis RW, et al. Clinic reported facilitators and barriers to pediatric cancer survivor care delivery among survivorship clinics: A fishbone analysis. Pediatric blood & cancer. 2023;70(8):e30480.

9. Fairall L, Petersen I, Zani B, Folb N, Georgeu-Pepper D, Selohilwe O, et al. Collaborative care for the detection and management of depression among adults receiving antiretroviral therapy in South Africa: study protocol for the CobALT randomised controlled trial. Trials. 2018;19(1):193.

10. Petersen I, Selohilwe O, Georgeu-Pepper D, Ras CJ, Zani B, Petrus R, et al. A collaborative care package for depression comorbid with chronic physical conditions in South Africa. BMC Health Serv Res. 2022;22(1).

11. Vousden N, Lawley E, Seed PT, Gidiri MF, Charantimath U, Makonyola G, et al. Exploring the effect of implementation and context on a stepped-wedge randomised controlled trial of a vital sign triage device in routine maternity care in low-resource settings. Implement Sci. 2019;14(1).

12. Almeida FA, Michaud TL, Wilson KE, Schwab RJ, Goessl C, Porter GC, et al. Preventing diabetes with digital health and coaching for translation and scalability (PREDICTS): A type 1 hybrid effectiveness-implementation trial protocol. Contemporary Clinical Trials. 2020;88:105877.

13. Michaud TL, Wilson K, Silva F, Almeida F, Katula J, Estabrooks P. Costing a population health management approach for participant recruitment to a diabetes prevention study. Translational behavioral medicine. 2021;11(10):1864-74.

14. Goldstein KM, Fisher DA, Wu RR, Orlando LA, Coffman CJ, Grubber JM, et al. An electronic family health history tool to identify and manage patients at increased risk for colorectal cancer: protocol for a randomized controlled trial. Trials. 2019;20(1):576.

15. Voils CI, Coffman CJ, Wu RR, Grubber JM, Fisher DA, Strawbridge EM, et al. A Cluster Randomized Trial of a Family Health History Platform to Identify and Manage Patients at Increased Risk for Colorectal Cancer. J Gen Intern Med. 2023;38(6):1375-83.

16. Woodard L, Amspoker AB, Hundt NE, Gordon HS, Hertz B, Odom E, et al. Comparison of Collaborative Goal Setting with Enhanced Education for Managing Diabetes-Associated Distress and Hemoglobin A1cLevels: A Randomized Clinical Trial. JAMA Netw Open. 2022;5(5).

17. Vandermorris A, McKinnon B, Sall M, Witol A, Traore M, Lamesse-Diedhiou F, et al. Adolescents' experiences with group antenatal care: Insights from a mixed-methods study in Senegal. Tropical Medicine and International Health. 2021;26(12):1700-8.

18. Lewis MA, Wagner LK, Rosas LG, Lv N, Venditti EM, Steinman LE, et al. Using RE-AIM to examine the potential public health impact of an integrated collaborative care intervention for weight and depression management in primary care: Results from the RAINBOW trial. PLoS ONE. 2021;16(3 March).

19. Ma J, Yank V, Lv N, Goldhaber-Fiebert JD, Lewis MA, Kramer MK, et al. Research aimed at improving both mood and weight (RAINBOW) in primary care: A type 1 hybrid design randomized controlled trial. Contemporary clinical trials. 2015;43:260-78.

20. Venditti EM, Steinman LE, Lewis MA, Weiner BJ, Ma J. Seeking a pot of gold with integrated behavior therapy and research to improve health equity: insights from the RAINBOW trial for obesity and depression. Transl Behav Med. 2021;11(9):1691-8.

21. Hagedorn HJ, Stetler CB, Bangerter A, Noorbaloochi S, Stitzer ML, Kivlahan D. An implementation-focused process evaluation of an incentive intervention effectiveness trial in substance use disorders clinics at two Veterans Health Administration medical centers. Addiction science & clinical practice. 2014;9(101316917):12.

22. Smith LR, Perez-Brumer A, Nicholls M, Harris J, Allen Q, Padilla A, et al. A data-driven approach to implementing the HPTN 094 complex intervention INTEGRA in local communities. Implementation Science. 2024;19(1).

23. Meffert SM, Neylan TC, McCulloch CE, Blum K, Cohen CR, Bukusi EA, et al. Interpersonal psychotherapy delivered by nonspecialists for depression and posttraumatic stress disorder among Kenyan HIV–positive women affected by gender-based violence: Randomized controlled trial. PLoS Med. 2021;18(1).

24. Onu C, Ongeri L, Bukusi E, Cohen CR, Neylan TC, Oyaro P, et al. Interpersonal psychotherapy for depression and posttraumatic stress disorder among HIV-positive women in Kisumu, Kenya: study protocol for a randomized controlled trial. Trials. 2016;17(1):64.

25. Belus JM, Rose AL, Andersen LS, Ciya N, Joska JA, Myers B, et al. Adapting a Behavioral Intervention for Alcohol Use and HIV Medication Adherence for Lay Counselor Delivery in Cape Town, South Africa: A Case Series. Cogn Behav Pract. 2022;29(2):454-67.

26. Magidson JF, Joska JA, Belus JM, Andersen LS, Regenauer KS, Rose AL, et al. Project Khanya: results from a pilot randomized type 1 hybrid effectiveness-implementation trial of a peer-delivered behavioural intervention for ART adherence and substance use in HIV care in South Africa. Journal of the International AIDS Society. 2021;24(S2):e25720.

27. Magidson JF, Joska JA, Myers B, Belus JM, Regenauer KS, Andersen LS, et al. Project Khanya: a randomized, hybrid effectiveness-implementation trial of a peer-delivered behavioral intervention for ART adherence and substance use in Cape Town, South Africa. 2020;1.

28. Shepardson RL, Weisberg RB, Wade M, Maisto SA, Funderburk JS. Brief modular anxiety intervention for primary care: Hybrid I pilot randomized controlled trial of feasibility, acceptability, effectiveness, and implementation potential. Journal of Affective Disorders. 2024;361:497-507.

29. Arrossi S, Paolino M, Antelo VS, Thouyaret L, Kohler RE, Cuberli M, et al. Effectiveness of an mHealth intervention to increase adherence to triage of HPV DNA positive women who have performed self-collection (the ATICA study): A hybrid type I cluster randomised effectiveness-implementation trial. 2022;9.

30. Arrossi S, Paolino M, Orellana L, Thouyaret L, Kohler RE, Viswanath K. Mixed-methods approach to evaluate an mHealth intervention to increase adherence to triage of human papillomavirus-positive women who have performed self-collection (the ATICA study): study protocol for a hybrid type I cluster randomized effectiveness-implementation trial. Trials. 2019;20(1):148.

31. Paolino M, Sanchez Antelo V, Kohler RE, Viswanath K, Arrossi S. Implementation of an mHealth intervention to increase adherence to triage among HPV positive women with HPV-self-collection (ATICA study): post-implementation evaluation from the women's perspective. BMC women's health. 2023;23(1):332.

32. Straw C, Sanchez-Antelo V, Kohler R, Paolino M, Viswanath K, Arrossi S. Implementation and scaling-up of an effective mHealth intervention to increase adherence to triage of HPV-positive women (ATICA study): perceptions of health decision-makers and health-care providers. BMC health services research. 2023;23(1):47.

33. Smit MMC, Waal ED, Tenback DE, Deenik J. Evaluating the implementation of a multidisciplinary lifestyle intervention for people with severe mental illness in sheltered housing: effectiveness-implementation hybrid randomised controlled trial. BJPsych Open. 2022;8(6):e201.

34. Paulsen MM, Varsi C, Andersen LF. Process evaluation of the implementation of a decision support system to prevent and treat disease-related malnutrition in a hospital setting. BMC health services research. 2021;21(1):281.

35. Cabassa LJ, Stefancic A. Context before implementation: A qualitative study of decision makers’ views of a peer-led healthy lifestyle intervention for people with serious mental illness in supportive housing. Transl Behav Med. 2019;9(2):217-26.

36. Cabassa LJ, Stefancic A, Lewis-Fernández R, Luchsinger J, Weinstein LC, Guo S, et al. Main Outcomes of a Peer-Led Healthy Lifestyle Intervention for People With Serious Mental Illness in Supportive Housing. Psychiatr Serv. 2021;72(5):555-62.

37. Cabassa LJ, Stefancic A, O'Hara K, El-Bassel N, Lewis-Fernández R, Luchsinger JA, et al. Peer-led healthy lifestyle program in supportive housing: study protocol for a randomized controlled trial. Trials. 2015;16:388.

38. Tuda D, Bochicchio L, Stefancic A, Hawes M, Chen JH, Powell BJ, et al. Using the matrixed multiple case study methodology to understand site differences in the outcomes of a Hybrid Type 1 trial of a peer-led healthy lifestyle intervention for people with serious mental illness. Translational behavioral medicine. 2023((Tuda, Hawes, Chen, Powell, Cabassa) George Warren Brown School of Social Work, Washington University in St. Louis Campus Box 1196 ,One Brookings Drive, St. Louis, United States(Tuda, Hawes, Powell, Cabassa) Center for Mental Health Services Research, Bro).

39. Vest BM, Wray LO, Brady LA, Thase ME, Beehler GP, Chapman SR, et al. Primary care and mental health providers’ perceptions of implementation of pharmacogenetics testing for depression prescribing. BMC Psychiatry. 2020;20(1).

40. Garner BR, Burrus O, Ortiz A, Tueller SJ, Peinado S, Hedrick H, et al. A Longitudinal Mixed-Methods Examination of Positive Health Check: Implementation Results From a Type 1 Effectiveness-Implementation Hybrid Trial. Journal of acquired immune deficiency syndromes (1999). 2022;91(1):47-57.

41. Lewis MA, Harshbarger C, Bann C, Burrus O, Peinado S, Garner BR, et al. Positive Health Check evaluation: A type 1 hybrid design randomized trial to decrease HIV viral loads in patients seen in HIV primary care. Contemp Clin Trials. 2020;96:106097.

42. Lewis MA, Harshbarger C, Bann C, Marconi VC, Somboonwit C, Piazza MD, et al. Effectiveness of an Interactive, Highly Tailored "Video Doctor" Intervention to Suppress Viral Load and Retain Patients With HIV in Clinical Care: A Randomized Clinical Trial. Journal of acquired immune deficiency syndromes (1999). 2022;91(1):58-67.

43. Nelson LA, Roddy MK, Bergner EM, Gonzalez J, Gentry C, Lestourgeon LM, et al. Exploring determinants and strategies for implementing self-management support text messaging interventions in safety net clinics. J Clin Transl Sci. 2022;6(1).

44. Nelson LA, Wallston KA, Kripalani S, Greevy RA, Jr., Elasy TA, Bergner EM, et al. Mobile Phone Support for Diabetes Self-Care Among Diverse Adults: Protocol for a Three-Arm Randomized Controlled Trial. JMIR Res Protoc. 2018;7(4):e92.

45. Ellis DA, Carcone AI, Naar-King S, Rajkumar D, Palmisano G, Moltz K. Adaptation of an Evidence-Based Diabetes Management Intervention for Delivery in Community Settings: Findings From a Pilot Randomized Effectiveness Trial. J Pediatr Psychol. 2019;44(1):110-25.

46. Frost J, Wingham J, Britten N, Greaves C, Abraham C, Warren FC, et al. The value of social practice theory for implementation science: learning from a theory-based mixed methods process evaluation of a randomised controlled trial. BMC medical research methodology. 2020;20(1):181.

47. Brady JE, Livingston NA, Sawdy M, Yeksigian K, Zhou S, Bickmore TW, et al. Development and Evaluation of a Relational Agent to Assist with Screening and Intervention for Unhealthy Drinking in Primary Care. J technol behav sci. 2023.

48. Rubin A, Livingston NA, Brady J, Hocking E, Bickmore T, Sawdy M, et al. Computerized Relational Agent to Deliver Alcohol Brief Intervention and Referral to Treatment in Primary Care: a Randomized Clinical Trial. J Gen Intern Med. 2022;37(1):70-7.

49. Ventuneac A, Li DH, Mongrella MC, Moskowitz DA, Weingardt KR, Brown CH, et al. Exploring Potential Implementation Barriers and Facilitators of the SMART Program: A Stepped-Care Package of eHealth HIV Prevention Interventions for Adolescent Men Who Have Sex with Men. Sex Res Soc Policy. 2020;17(3):378-88.

50. Binns E, Bright F, Parsons J, Peri K, Taylor L, Kerse N, et al. “It’s all about the money”: an interpretive description of embedding physical therapy-led falls prevention group exercise in long-term care. BMC Geriatr. 2023;23(1).

51. Reckrey JM, Gazarian P, Reuben DB, Latham NK, McMahon SK, Siu AL, et al. Barriers to implementation of STRIDE, a national study to prevent fall-related injuries. J Am Geriatr Soc. 2021;69(5):1334-42.

52. Smith JD, Stormshak EA, Kavanagh K. Results of a pragmatic effectiveness-implementation hybrid trial of the Family Check-up in community mental health agencies. Administration and policy in mental health. 2015;42(3):265-78.

53. Minian N, Lingam M, Moineddin R, Thorpe KE, Veldhuizen S, Dragonetti R, et al. Impact of a Web-Based Clinical Decision Support System to Assist Practitioners in Addressing Physical Activity and/or Healthy Eating for Smoking Cessation Treatment: Protocol for a Hybrid Type I Randomized Controlled Trial. JMIR Res Protoc. 2020;9(9):e19157.

54. Minian N, Lingam M, Moineddin R, Thorpe KE, Veldhuizen S, Dragonetti R, et al. The Impact of a Clinical Decision Support System for Addressing Physical Activity and Healthy Eating During Smoking Cessation Treatment: Hybrid Type I Randomized Controlled Trial. Journal of medical Internet research. 2022;24(9):e37900.

55. Dawson R, Gilchrist H, Pinheiro M, Nelson K, Bowes N, Sherrington C, et al. Experiences of Older Adults, Physiotherapists, and Aged Care Staff in the TOP UP Telephysiotherapy Program: interview Study of the TOP UP Interventions. JMIR aging. 2024;7:e53010.

56. Dawson R, Pinheiro M, Nagathan V, Taylor M, Delbaere K, Olivera J, et al. Physiotherapy-led telehealth and exercise intervention to improve mobility in older people receiving aged care services (TOP UP): protocol for a randomised controlled type 1 hybrid effectiveness-implementation trial. BMJ nutrition, prevention & health. 2023;6(2):273‐81.

57. Elwy AR, Kim B, Plumb DN, Wang S, Gifford AL, Asch SM, et al. The Connectedness of Mental Health Providers Referring Patients to a Treatment Study for Post-Traumatic Stress: A Social Network Study. Administration and policy in mental health. 2020;47(2):197-209.

58. Purcell N, Becker WC, Zamora KA, McGrath SL, Hagedorn HJ, Fabian ER, et al. Tailored to Fit: How an Implementation Framework Can Support Pragmatic Pain Care Trial Adaptation for Diverse Veterans Affairs Clinical Settings. Med Care. 2020;58:S80-S7.

59. Holle D, Roes M, Buscher I, Reuther S, Müller R, Halek M. Process evaluation of the implementation of dementia-specific case conferences in nursing homes (FallDem): study protocol for a randomized controlled trial. Trials. 2014;15:485.

60. Teupen S, Holle D, Roes M. Types of implementation of the dementia-specific case conference concept WELCOME-IdA in nursing homes: a qualitative process evaluation of the FallDem effectiveness trial. Implement Sci Commun. 2021;2(1).
