# Supplementary material for: The use of implementation science theoretical approaches in hybrid effectiveness-implementation type 1 randomised trials of healthcare interventions: A scoping review
Source: Implement Sci. 2025 May 16;20:23. doi: 10.1186/s13012-025-01435-6 (PMC12083051; doi:10.1186/s13012-025-01435-6)
Supplement: Supplementary file 6 — Additional file 6. [file 13012_2025_1435_MOESM6_ESM.docx]

Extent of use of TMFs in included trials

| **Author, year, reference** | **Theory/Model/Framework** | **Type of Application/Informed** | **Illustrative quote/s [page no]** |
| --- | --- | --- | --- |
| **APPLIED** | | | |
| Arrossi et al^(37)^ Straw et al ^(40)^ | RE-AIM | To select study materials and analyse data | "Individual semi-structured interviews were conducted using a guide based on included constructs of CFIR and RE-AIM." [pg 4]. "The themes explored were selected and analyzed using the domains and constructs of the Consolidated Framework for Implementation Research (CFIR), and the maintenance dimension of the RE-AIM". [pg 3]  "RE-AIM framework was integrated in all stages of the research process, including conceptualization (e.g., selecting implementation processes that would be evaluated), data collection and analysis" [pg 4] |
| Baloh et al ^(42)^ | CFIR | To select study materials and analyse data | "The semi-structured interview guide, based on the Consolidated Framework for Implementation Research, guided data collection on implementation determinants from five domains: intervention characteristics ..., outer setting ..., inner setting ..., staff characteristics ..., and process..." [pg 2] "We thematically analyzed the interviews using both deductive (... and domains of the Consolidated Framework for Implementation Research) and inductive (emergent codes) approaches". [pg 2] |
| Cabassa and Stefancic ^(25)^ | CFIR | To select study materials and analyse data | "The interview guide was informed by …. the Consolidated Framework for Implementation Research (CFIR) to inquire about participants’ views of PGLB and contextual factors known to influence the implementation process in their specific agencies." [pg 219]  "The interview included open-ended questions... Each factor was derived from the CFIR and included: outer-setting system-level factors ..., and inner-setting factors composed of organization-level factors ..., intervention characteristics ..., staff-level factors ..., and client-level factors ...." [pg 219] "To develop our initial codes, we started with key concepts derived from the Diffusion of Innovations Theory and the CFIR in terms of intervention characteristics and contextual factors at the outer and inner settings." [pg 219] |
| Cabassa and Stefancic ^(25)^ | Theory of diffusion of innovations | To select study materials and analyse data | "The interview guide ... was informed by constructs derived from the Diffusion of Innovations Theory, particularly innovation attributes..." [pg 219]  "To develop our initial codes, we started with key concepts derived from the Diffusion of Innovations Theory and the CFIR in terms of intervention characteristics and contextual factors at the outer and inner settings." [pg 219] |
| Lewis et al ^(45)^ | RE-AIM | To select study materials and analyse data | "Using a Framework Analysis approach, we examined themes across time and stakeholder groups according to the RE-AIM framework" [pg 1]  "...investigators created a codebook to define key themes and concepts using the RE-AIM framework" [pg 5] |
| Magidson et al ^(29)^ | Proctor's taxonomy of implementation outcome | To select study materials and analyse data | "Implementation outcomes were guided by Proctor’s model, including feasibility, acceptability, appropriateness and fidelity." [pg 119] |
| Minian et al ^(33)^ | RE-AIM | To select study materials and analyse data | "The RE-AIM framework was used to measure implementation outcomes in the intervention group" [pg 3]  "We used the RE-AIM framework to structure and interpret the study outcomes." [pg 3] |
| Nelson et al ^(46)^ | CFIR | To select study materials and analyse data | "Interview questions were developed using the Consolidated Framework for Implementation Research (CFIR)" [pg 2]  "All 5 CFIR domains were represented across each interview guide." [pg 3] "Using an open coding approach (inductive approach), we first identified themes and then classified them as barriers and facilitators. Next, we mapped CFIR constructs onto the coded data (deductive approach). Codebooks were developed for patient and staff interviews based on both initial open coding of transcripts, the CFIR-informed interview guide, and definitions of CFIR constructs." [pg 3] |
| Paulsen et al ^(26)^ | RE-AIM | To select study materials and analyse data | "The RE-AIM framework was used to guide the process evaluation... the five RE-AIM dimensions: reach, effectiveness, adoption, implementation and maintenance, and how they were assessed during the process evaluation in the randomized controlled trial using both quantitative and qualitative methods" [pg 3] "... initial codes were created. Third, themes were established deductively for the RE-AIM dimension ‘Implementation'." [pg 5] |
| Reckrey et al ^(47)^ | CFIR | To select study materials and analyse data | "A semi-structured interview guide derived from the consolidated framework for implementation research (CFIR)." [pg 1334]  "While the codebook placed emphasis on deductive or a priori codes corresponding to the defined CFIR domains and constructs, we also used inductive approaches to create codes that described new themes not adequately captured in the CFIR framework." [pg 1337] |
| Smith et al ^(51)^ | PRISM | To select study materials and analyse data | "The implementation evaluation is guided by the PRISM framework ... PRISM’s four theoretical domains guided our implementation assessments of (i) the patient ... and healthcare organizational... perceptions of INTEGRA’s integrated evidence-based interventions ..., (ii) characteristics of PWID and the mobile unit staff that affect the delivery of integrated care, (iii) factors in the external community environment that can influence how integrated care was delivered by the mobile unit and accessed by PWID, and (iv) systems-level factors and infrastructure needs that affect or are affected by the delivery of integrated care through the mobile unit." [pg 3] "Analysis was guided by PRISM and the quantitative data was presented on maps via color-coding to identify high-enrollment neighborhoods and the degree to which new neighborhoods were added during the implementation phase... This was an inductive and deductive approach as PRISM guided it to facilitate the ability to compare and contrast observations within and across sites while also learning from the unique contextual factors affecting neighborhood viability." [pg 5] |
| Straw et al ^(40)^ | CFIR | To select study materials and analyse data | "Individual semi-structured interviews were conducted using a guide based on included constructs of CFIR and RE-AIM." [pg 4]. "The themes explored were selected and analyzed using the domains and constructs of the Consolidated Framework for Implementation Research (CFIR), and the maintenance dimension of the RE-AIM". [pg 3] |
| Vest et al ^(52)^ | CFIR | To select study materials and analyse data | "Focus group questions were developed based on CFIR Intervention Characteristics constructs, including: Intervention Source, Evidence, Relative Advantage, Adaptability, Trialability, Complexity, Intervention Design, and Cost. " [pg 3] "Each theme was then coded by CFIR construct." [pg 4] |
| Woodard et al ^(41)^ | RE-AIM | To select study materials and analyse data | "Secondary implementation outcomes evaluated the remaining RE-AIM dimensions: (1) reach was the number of eligible patients who participated in the study; (2) adoption was the proportion of actual vs planned EPICC sessions conducted by trained clinicians; and (3) implementation was the number of group sessions attended per patient and how this number is associated with primary outcomes." [pg 5] |
| Dawson et al ^(35)^ | COM-B | To select study materials and analyse data | "We used 2 behavior change theories, COM-B and SDT, and the Nonadoption, Abandonment and Challenges to the Scale-Up, Spread and Sustainability of Health and Care Technologies (NASSS) framework to provide a conceptual “lens” to inform data collection and analysis" [pg 3] "Recurrent themes were generated from reading across the coded data and reviewed against concepts from SDT and the COM-B framework to understand how aspects of the TOP UP program influenced exercise engagement. " [pg 5] |
| Dawson et al ^(35)^ | Self-Determination Theory | To select study materials and analyse data | "We used 2 behavior change theories, COM-B and SDT, and the Nonadoption, Abandonment and Challenges to the Scale-Up, Spread and Sustainability of Health and Care Technologies (NASSS) framework to provide a conceptual “lens” to inform data collection and analysis" [pg 3] "Recurrent themes were generated from reading across the coded data and reviewed against concepts from SDT and the COM-B framework to understand how aspects of the TOP UP program influenced exercise engagement. " [pg 5] |
| Dawson et al ^(35)^ | Nonadoption, Abandonment and Challenges to the Scale-Up, Spread and sustainability of Health and Care Technologies | To select study materials and analyse data | "We used 2 behavior change theories, COM-B and SDT, and the Nonadoption, Abandonment and Challenges to the Scale-Up, Spread and Sustainability of Health and Care Technologies (NASSS) framework to provide a conceptual “lens” to inform data collection and analysis" [pg 3] "Recurrent themes were generated from reading across the coded data and reviewed against concepts from SDT and the COM-B framework to understand how aspects of the TOP UP program influenced exercise engagement. " [pg 5] |
| Paolino et al ^(39)^ | Proctor's taxonomy of implementation outcome | To select study materials | "Following Proctor ́s Taxonomy of Implementation Outcomes, we measured acceptability and appropriateness, which are essential to understand the success (or failure) of the implementation of an intervention from users’ perspective". [pg 3] |
| Hagedorn et al ^(28)^ | RE-AIM | To select study materials | "Guided by [RE-AIM] and [PARIHS] framework we developed a series of questions that would provide valuable information for future implementation efforts" [pg 3] |
| Hagedorn et al ^(28)^ | PARiHS | To select study materials | "Guided by [RE-AIM] and [PARIHS] framework we developed a series of questions that would provide valuable information for future implementation efforts" [pg 3] |
| Krauss et al ^(44)^ | Socio-ecological model | To select study materials | "... focused on questions regarding staff and provider’s perceptions of cancer survivors’ knowledge about cancer survivorship, and facilitators and barriers related to engagement in survivor care at all levels of the SEM framework: individual and intrapersonal, organizational, community, and societal/policy " [pg 2] |
| Ventuneac et al ^(34)^ | RE-AIM | To select study materials | "Development of the interview questions was guided by the RE-AIM framework to address the following domains: 1) reach 2) effectiveness or efficacy 3) adoption 4) implementation and 5) sustainability" [pg 381] |
| Ventuneac et al ^(34)^ | CFIR | To select study materials | "We also utilized the [CFIR] to supplement our evaluation with organization-level measures about the settings at which the SMART program could be implemented" [pg 381] |
| Voils et al ^(60)^ | Organisation Readiness for Change Theory | To select study materials | "... a structured interview guide that incorporated organizational-level implementation determinants to understand how PCPs value FHH for clinical decision-making (innovation-values fit23); how they became aware of their patients completing the FHH platform; how they used or did not use the CDS recommendations and any barriers to doing so (innovation-task fit24); how their patients reacted to the recommendations (implementation acceptability25); how receiving CDS reports and patient discussions affected their workflow; and the time required to act on the recommendations" [pg 1379] |
| Vousden et al ^(53)^ | RE-AIM | To select study materials | "The implementation and impact of the intervention in each site was evaluated by mixed-methods under three implementation domains … informed by the RE-AIM framework" [pg 4] |
| Boden et al ^(43)^ | RE-AIM | To justify study design and select study materials | "The study had two primary aims: ...., as guided by the reach, effectiveness, adoption, implementation, maintenance (RE-AIM ) framework" [pg 2]  "We conducted 30–60 min semi-structured interviews using the RE-AIM (reach, efficacy, adoption, implementation and maintenance) framework and the RE-AIM Planning Tool to identify barriers and facilitators to patient participation in ACT and implementation of ACT." [pg 2] |
| Magidson et al ^(30)^ | RE-AIM | To justify study design and select study materials | "This study is using a hybrid type 1 effectiveness-implementation design, guided by RE-AIM... " [pg 2]  "Qualitative interviews with providers and organizational leadership based on RE-AIM to assess perceptions and likelihood of uptake following the trial" [pg 3] |
| Almeida et al^(31),^ Michaud et al ^(32)^ | iPARiHS | To justify study design and select study materials | "The RE-AIM framework is used to guide the design of the PREDICTS HEI trial with a goal to plan explicitly and quantitatively address implementation outcomes related to reach and to qualitatively assess potential for organizational adoption and implementation" [pg 2] " Key informant interviews will be conducted consistent with the iPARIHS framework to obtain physician and administrator perceptions" [pg 4] |
| Almeida et al ^(31),^ Michaud et al ^(32)^ | RE-AIM | To justify study design, select study materials and analyse data | "The RE-AIM framework is used to guide the design of the PREDICTS HEI trial with a goal to plan explicitly and quantitatively address implementation outcomes related to reach and to qualitatively assess potential for organizational adoption and implementation" [pg 2] |
| Purcell et al ^(49)^ | PARiHS | To justify the study design | "Informed by the PARiHS framework, we gathered site-specific information relevant to wHOPE study implementation. The PARiHS framework requires attention not only to existing evidence (the scientific rationale for intervention implementation), but also to context (various local contextual factors likely to affect implementation) and to facilitation (strategic approaches to guiding/supporting local implementation). Our formative evaluation incorporated each of these elements." [pg 81] |
| Chlebowski et al ^(27)^ | FRAME | To analyse data | "Recommendations were provided in two domains informed by categories in the FRAME model… the application of the FRAME model to categorize specific therapist and parent recommendations..." [page 1203] |
| Teupen et al ^(69)^ | CFIR | To analyse data | "The three selected domains of the CFIR were used as deductive categories for a directed content analysis." [pg 4] |
| **INFORMED BY** |  |  |  |
| Sommerfeld et al ^(70)^ | EPIS | Informed study objectives and explanation of results | "EPIS identifies structural characteristics such as system-level policies (i.e., outer context) and organizational characteristics (i.e., inner context) as well as the process of implementation likely to be important for effectively implementing EBPs in organizations" [pg 34]  “In keeping with the EPIS framework, the results suggest that it is important to consider sustainment and not just implementation as any EBP implementation effort begins “ [pg 38] |
| Frost et al ^(54)^ | Social Practice Theory | Informed study objectives | "We used Social Practice Theory to guide a Type 1 Hybrid Trial: a mixed methods process evaluation of a complex intervention for heart failure. The objective of this paper is to explore the value of Social Practice Theory for implementation science." [pg 1] |
| Minian et al ^(33)^ | Interactive Systems Framework | Informed study objectives | "To better equip health care practitioners to implement the intervention, we undertook several knowledge translation initiatives based on the principles of the Interactive Systems Framework (ISF) for dissemination and implementation." [pg 3] |
| Paulsen et al ^(26)^ | CFIR | Informed study objectives | "The first phase of the implementation plan involved the assessment of potential barriers and facilitators of using [the intervention] in the hospital department, using the CFIR" [pg 3] |
| Teupen et al ^(69)^ | Grant et al. (2013) framework | Informed study objectives | "...a multipart process evaluation was conducted in parallel to the effectiveness trial following Grant et al.’s framework." [pg 3] |
| Petersen et al ^(55)^ | RE-AIM | Informed explanation of results | "We used the additional element of ‘actual implementation’ to identify actual implementation of the package and ‘modifications made’ across the two sites together with the RE-AIM framework. Notably, we focused on the reach and adoption of the training provided to the various providers in the system; as well as reach and adoption of the package in relation to patients as part of routine services. ‘Modifications made’ enabled us to report on the implementation element of the RE-AIM framework in relation to adaptations made across the two sites." [pg 4] |
| Elwy et al ^(48)^ | Theory of Diffusion of innovations | Informed intervention | **"**The social network survey development was guided by the Theory of Diffusion of Innovations" [pg 199] |
| **CITED** |  |  |  |
| Chlebowski et al ^(27)^ | Proctor's taxonomy of implementation outcome |  | "... a number of key implementation outcomes (Proctor et al., 2011) are important to understanding and informing adaptations... Relatedly, the acceptability of the intervention, which indexes whether one finds an intervention agreeable or satisfactory, may inform adaptations and is commonly measured via qualitative methods (Proctor et al., 2011)." [pg 1195] |
| Vandermorris et al ^(50)^ | Proctor's taxonomy of implementation outcomes |  | "Six key implementation outcomes were defined for the pilot study, based on a conceptual framework for implementation research" [pg 1702] |

*Note: Table is adapted from Colquhoun ^(11)^ and McIntyre et al ^(12)^*. *Arrossi et al^(37)^, Paolino et al^(39)^and Straw et al^(40)^are publications related to the mHealth intervention Arrossi et al^(38)^*
